# Dietary pH Enhancement Improves Metabolic Outcomes in Diet-Induced Obese Male and Female Mice: Effects of Beef vs. Casein Proteins

**DOI:** 10.3390/nu14132583

**Published:** 2022-06-22

**Authors:** Kalhara R. Menikdiwela, João Pedro Tôrres Guimarães, Shane Scoggin, Lauren S. Gollahon, Naima Moustaid-Moussa

**Affiliations:** 1 Laboratory of Nutrigenomics, Inflammation and Obesity Research, Department of Nutritional Sciences, Texas Tech University (TTU), Lubbock, TX 79409-1270, USA; kalhara.menikdiwela@ttu.edu (K.R.M.); jtorresg@ttu.edu (J.P.T.G.); shane.scoggin@ttu.edu (S.S.); 2 Obesity Research Institute, Texas Tech University, Lubbock, TX 79409-1270, USA; lauren.gollahon@ttu.edu; 3 Department of Biological Sciences, Texas Tech University, Lubbock, TX 79409-3131, USA

**Keywords:** dietary fat, obesity, beef, casein, pH, mice, males, females

## Abstract

(1) Consumption of diets that are caloric dense but not nutrient dense have been implicated in metabolic diseases, in part through low-grade metabolic acidosis. Mitigation strategies through dietary intervention to alleviate acidosis have not been previously reported. Our objective is to determine the effects of pH enhancement (with ammonia) in high fat diet-induced obese mice that were fed beef or casein as protein sources compared to low fat diet-fed mice. (2) Methods: B6 male and female mice were randomized (*n* = 10) into eight diets that differ in protein source, pH enhancement of the protein, and fat content, and fed for 13 weeks: low fat (11% fat) casein (LFC), LF casein pH-enhanced (LFCN), LF lean beef (LFB), LFBN, high fat (46%) casein (HFC), HFCN, HF beef (HFB), and HFBN. Body weights and composition, and glucose tolerance tests were conducted along with terminal serum analyses. Three-way ANOVA was performed. (3) Results: A significant effect of dietary fat (LF vs. HF) was observed across all variables in both sexes (final body weight, fat mass, glucose clearance, and serum leptin). Importantly, pH enhancement significantly reduced adiposity (males only) and final body weights (females only) and significantly improved glucose clearance in both sexes. Lastly, clear sex differences were observed across all variables. (4) Conclusions: Our findings demonstrate metabolic benefits of increasing dietary pH using ammonia, while high fat intake per se (not protein source) is the major contributor to metabolic dysfunctions. Additional research is warranted to determine mechanisms underlying the beneficial effects of pH enhancement, and interactions with dietary fat content and proteins.

## 1. Introduction

The prevalence of non-communicable diseases (NCDs) such as obesity, type 2 diabetes (T2D), cardiovascular diseases (CVDs) and certain cancers including breast cancer is increasing at a frightening rate in Western societies [1,2,3,4]. While many factors contribute to these diseases including genetics and gene-environment interactions, among others, chronic overnutrition of high caloric Western-type diets (WD) coupled with a sedentary lifestyle are important contributors to the progression of many of these NCDs. Specifically, the WD characterized by high amounts of saturated fats, processed foods and meats, sugar-sweetened foods/beverages and low consumption of fruits, vegetables, and lean proteins, have been implicated in metabolic diseases [5,6,7,8]. 

Long-term consumption of such WDs rich in fatty and processed foods and meats can influence metabolic impairments by promoting a variety of poor health outcomes [8,9,10,11,12], including pathological changes in lipid and energy metabolism and immune system activation of inflammatory responses [7,13]. Metabolic regulation and systemic immune responses are highly integrated, and diet mediated disruptions in immune-metabolic homeostasis can lead to the development of chronic diseases [8,11,12,13]. 

A potential mechanism through which immune-metabolic homeostasis is disrupted could be through low-grade metabolic acidosis. Diet is one of the key factors that influences metabolic acidosis (e.g., a slight decrease of pH from the lower limit of blood pH 7.35) [14,15]. Increased dietary acid load due to overconsumption of certain foods high in salt and fatty and processed meats (typical WD) could influence small changes in the acid-base balance (increase in [H^+^] and reduction in [HCO_3_^−^]) [14,16,17,18]. Although diet-induced low-grade metabolic acidosis causes slight changes in the body’s acid-base balance, the long-term impact of metabolic acidosis on metabolism and overall cellular homeostasis could be detrimental [17,19,20]. 

A plethora of evidence over the past few decades has revealed that persistent, low-grade metabolic acidosis could cause chronic diseases including obesity, T2D, and vascular diseases [19,20,21]. In general, animal-derived foods, including red meat (beef, pork), release higher numbers of precursors of acids into the bloodstream, as indicated by a higher potential renal acid load (PRAL) value [5,17,22]. Beef is one of the most highly consumed red meats in the United States of America and is rich in essential nutrients (proteins, iron) [23,24,25]. Consumer-ready beef pH lies in slightly acidic range (pH 5.3 to 5.7) [26,27]. This pH range might even further affected by meat processing, preparation and conventional cooking methods, changing the acid load [18,28,29] and in turn, contributing to metabolic impairments [30]. Additionally, increased red meat intake may be associated with higher prevalence of NCDs, including T2D and CVDs [31,32]. However, limited studies are available to support this argument [31,32], and several knowledge gaps exist in our understanding of the role of dietary pH, specifically in relation to protein, thus warranting additional research in this area. Moreover, it is not well understood whether the fat content of the diet, or the protein source per se, more greatly affects metabolic homeostasis within the body, contributing to NCDs. 

Hence, our objective for the current study was to test the effects of pH, fat content and the source of protein in low fat (LF), as well as high fat (HF) diet-induced obese B6 male and female mice. We hypothesized that metabolic health will be improved by consuming a diet containing pH-enhanced beef or casein, compared to non pH-enhanced beef or casein diets, and that metabolic responses will be sex-dependent. A novel contribution of this research is determining the metabolic and sex-dependent effects of dietary pH with two different protein sources (beef and casein), which have not been previously investigated.

## 2. Materials and Methods

**Figure 1:**
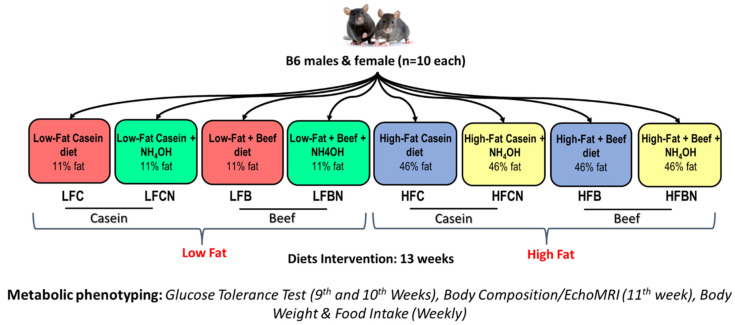
Study design and the classification of different groups. LFC = low fat casein; LFCN = low fat pH enhanced casein; LFB = low fat beef; LFBN = low fat pH enhanced beef; HFC = high fat casein; HFCN = high fat pH enhanced casein; HFB = high fat beef; HFBN = high fat pH enhanced beef.

**Animal study and diets:** Male and female virgin C57BL/6J mice, aged 4–5 weeks old, were purchased from Jackson Laboratory (Bar Harbor, ME, USA). After one-week acclimatization, male and female B6 mice were randomized into 8 customized diets (10 mice per each group: total 160 mice) based on the protein source (either beef protein: B or casein: C), pH enhancement (through beef or casein treatment with ammonia, prior to incorporation of these protein sources into mouse diets) and dietary fat content (low fat:11% fat kcals %, or high fat: 46% fat kcals %): (i) low fat (11% fat kcals %) casein (LFC), (ii) LF diet with pH-enhanced casein (ammonia treated: N) (LFCN), (iii) low fat lean beef (LFB), (iv) LF diet with pH-enhanced beef (ammonia treated) (LFBN), (v) high fat (46% fat kcals %) casein (HFC), (vi) HF diet with pH-enhanced casein (HFCN), (vii) HF with beef (HFB), and (viii) HF diet with pH-enhanced beef (HFBN). Mice were maintained on these diets (in the pellet form) throughout the study. Cooked ground beef was kindly provided to us by Empirical Foods, Inc (North Sioux City, SD, USA). Nutritional analyses of the beef products were conducted by an independent laboratory (Eurofins Scientific Inc. Des Moines, IA, USA), and diets were prepared based on these analyses, by Research Diets Inc (New Brunswick, NJ, USA). Detailed diet composition is provided in Supplemental Table S1 and the overall study design in Figure 1. 

Mice had access to the respective diets and water ad libitum. The mice were housed in ventilated cages individually with corn cob bedding at 22–23 °C and 70% humidity for the duration of the study (13 weeks). All the animal rooms were maintained on 12-h day/night with light onset at 7:00 am. Mice weights and food intake were measured weekly. At the end of the dietary interventions, mice were fasted for 5 h before euthanasia by CO_2_ asphyxiation, followed by exsanguination via cardiac puncture. All animal protocols were approved by the Institutional Animal Care and Use Committee of Texas Tech University (protocol number 21023-03). 

**Glucose Tolerance Test (GTT):** GTTs were performed on male and female mice in the 9th and 10th weeks, respectively, of dietary intervention. Mice were fasted for 5 h from 8:00 am to 1:00 pm. Tail blood was drawn for baseline measurement using a glucometer (Zoetis, Parsippany, NJ, USA). Mice were then given 2 g glucose/kg of body weight (20% glucose), intraperitoneally [33]. Glucose was measured at 30-, 60-, 90-, and 120-min intervals. In this system, the maximal level of blood glucose that could be determined was 700 mg/dL. The area under the curve (AUC) of GTT was assessed and calculated according to the trapezoidal method as previously reported [33]. 

**Body composition:** Magnetic resonance imaging (MRI) was used to assess body composition (fat mass and lean mass) using an Echo-MRI 3-in-1 analyzer (EchoMRI LLC, Houston, TX, USA) at the 10th and 11th weeks (for male and female mice, respectively) of the dietary interventions. 

**Serum Measurement:** Serum levels of insulin and leptin were measured using a MILLIPLEX MAP Mouse Adipocyte Magnetic Panel (MADKMAG-71K) (Millipore, Darmstadt, Germany) according to manufacturer’s instructions from the Luminex multiplexing system (Luminex xMAP, Austin, TX, USA). 

**Statistical Analyses:** Using Prism (GraphPad Software 9.2.0, San Diego, CA, USA), data were analyzed by performing three-way analysis of variance (ANOVA), including a main effect for pH enhancement (not treated or ammonia treated), protein source (casein or beef protein), and diet fat content (LF or HF) and their interactions. To identify whether there was a sex effect, three-way ANOVA was again performed including a main effect for sex (male and female), pH enhancement (not treated or ammonia treated), and protein source (casein or beef protein) separately for LF groups and HF fed groups. If significant, Tukey-corrected post hoc pairwise comparisons were made, maintaining a Family-wise error rate at 0.05. Results are presented as mean ± SEM (standard error of mean), with statistical significance considered at *p* < 0.05.

## 3. Results

### 3.1. Food Intake of Male and Female Mice during Dietary Intervention

**Figure 2:**
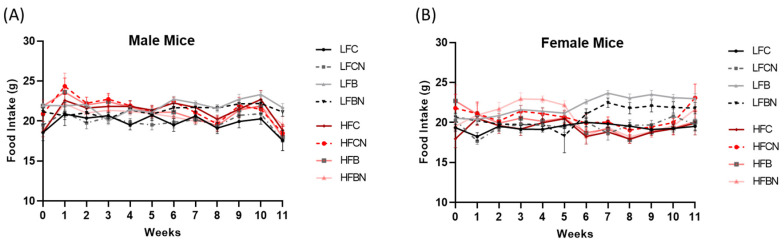
Weekly food consumption in (**A**) male and (**B**) female mice throughout the dietary intervention. LFC, low fat casein; LFCN, low fat casein pH enhanced; LFB, low fat beef; LFBN, low fat beef pH enhanced; HFC, high fat casein; HFCN, high fat casein pH enhanced; HFB, high fat beef; HFBN, high fat beef pH enhanced. *p* < 0.05; *n* = 8–10.

**Figure 3:**
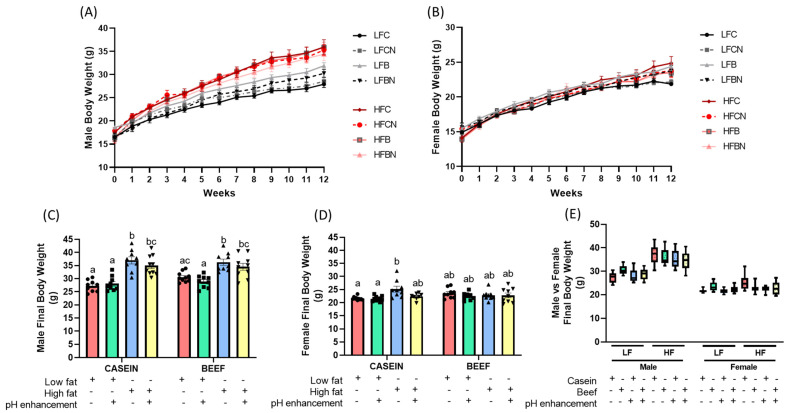
Sex-dependent effects of diet, protein source and pH enhancement on weight gain, and final body weight in diet induced obese male and female mice. Growth curves during the dietary interventions (**A**) male, (**B**) female mice, and the final body weight (**C**) male and (**D**) female mice. (**E**) Final body weight male vs. female mice. Data is presented as mean ± SEM. LFC, low fat casein; LFCN, low fat casein pH enhanced; LFB, low fat beef; LFBN, low fat beef pH enhanced; HFC, high fat casein; HFCN, high fat casein pH enhanced; HFB, high fat beef; HFBN, high fat beef pH enhanced. Common letters on the error bars indicate no significance (e.g., “a” is significantly different from “b” while “ab” indicates no significance compared to “a” and “b”). *p* < 0.05; *n* = 8–10.

In male mice, no significant changes in food intake were observed across treatment groups during the study period (Figure 2A). However, in female mice, LFB and LFBN groups indicated a significantly higher food intake during the 7th, 8th and 9th weeks compared to all the HF fed groups including HFC, HFCN, HFB and HFBN (Figure 2B). Interestingly, although LFB and LFBN groups demonstrated a higher food consumption during later parts of the dietary intervention, body weights were not significantly increased in LFB and LFBN groups in females (Figure 3B). On the other hand, in male mice, while their food intake was similar across all treatment groups, HF fed mice exhibited significantly higher weight gain compared to LF fed mice regardless of the protein source and pH enhancement (Figure 3A).

### 3.2. Sex-Dependent Effects of Diet, Protein Source and pH Enhancement on Weight Gain, Fat Mass and Lean Mass

In males, three-way ANOVA revealed that HF diet significantly increased final body weight (F (1, 67) = 90.27, *p* < 0.0001), fat mass (F (1, 39) = 55.64, *p* < 0.0001) and lean mass (F (1, 41) = 4.512, *p* < 0.0397) compared to LF fed male mice (Table 1), indicating clear main effects of diet (LF vs. HF) on weight related variables in male. Additionally, significant effects of protein source on fat mass (F (1, 39) = 8.517, *p* < 0.0058) and lean mass (F (1, 41) = 7.151, *p* < 0.0107) in male mice were observed, with no significant effects of protein source on final body weights (Table 1), suggesting a potential role of beef protein in increasing muscle mass over casein protein (Table 1). Although, the pH enhancement with ammonia did not significantly change final body weights, pH enhancement significantly reduced fat mass compared to non pH enhanced male mice (F (1, 39) = 4.996, *p* < 0.0312) (Table 1). The effect of pH in reducing fat mass was prominent in HF fed males where pH enhancement reduced ≈ 20% of fat mass compared to non-pH enhanced fat mass in males. Interestingly, no significant interactions between factors (protein source; P, diet; D and pH; H) were observed in male mice for final body weight or fat mass among various diets (Table 1). However, significant interactions between diet and pH (D × H) for lean mass was observed in male mice (Table 1).

Although observed changes were small in females, female mice also demonstrated significantly higher final body weight (F (1, 64) = 4.578, *p* < 0.0362) and fat mass (F (1, 65) = 14.96, *p* < 0.0003) in HF fed females compared to LF groups, with no diet effect on lean mass (Table 2). However, in female mice, neither casein nor beef as the protein source significantly affected the final body weight, fat mass or the lean mass (Table 2). Yet, female mice exhibited a significant main effect of pH enhancement on final body weight (F (1, 64) = 4.952, *p* < 0.0296) indicating a potential role of increased dietary pH in reducing body weight (≈5%) (Table 2). Moreover, three-way ANOVA further revealed a significant interaction between protein and diet (P × D) for final body wight and fat mass in female mice (Table 2). No interaction was observed between protein and pH enhancement (P × H) for body weights. However, a significant interaction between diet and pH (D × H) was observed for fat mass in female mice (Table 2). A significant interaction was also noted in all three factors (protein, diet and pH; P × D × H) for final body weight for female mice (Table 2). 

A clear obesogenic effect of HF diets was observed in male body weight over the course of dietary intervention. The multiple comparison post hoc test with Tukey correction demonstrated that all four HF fed groups (HFC, HFCN, HFB, and HFBN) exhibited a considerably higher weight (*p* < 0.05) compared to LFC (starting from 5th week till the end), LFCN (starting from 6th week till the end) and LFBN (starting from 7th week till the end) groups (*p* < 0.05) (Figure 3A and Supplemental Table S2A). However, all HF groups (HFN, HFB and HFBN) except for HFC groups demonstrated no significant changes in body weights compared to the male LFB group, where the HFC group showed significantly higher weights in 9th and 10th weeks compared to the LFB group (Figure 3A and Supplemental Table S2A). 

**Figure 4:**
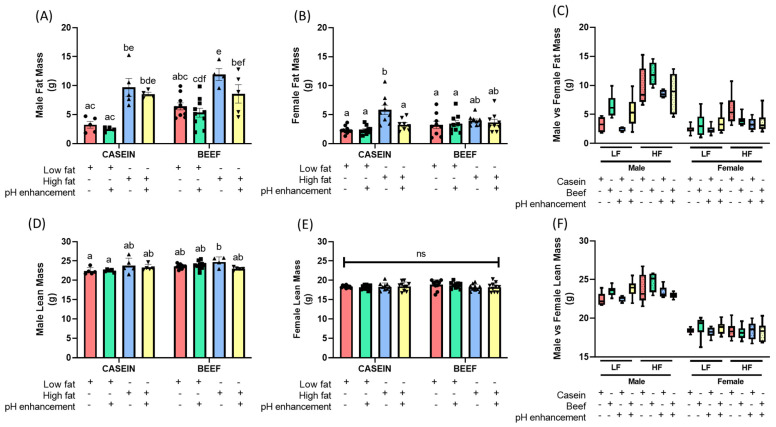
Effects of dietary fat, protein source, pH enhancement and sex in fat mass and lean mass in diet induced obese male and female mice. Fat mass (**A**) male and (**B**) female. Lean mass (**D**) male mice and (**E**) female mice were measured at the week 11 of the diet interventions. (**C**) Fat mass male vs. female mice and (**F**) lean mass male vs. female mice. Data is presented as mean ± SEM. LFC, low fat casein; LFCN, low fat casein pH enhanced; LFB, low fat beef; LFBN, low fat beef pH enhanced; HFC, high fat casein; HFCN, high fat casein pH enhanced; HFB, high fat beef; HFBN, high fat beef pH enhanced. Common letters on the error bars indicate no significance (e.g., “a” is significantly different from “b”; while “ab” indicates no significance compared to “a” and “b”). *p* < 0.05; *n* = 4–10.

Interestingly, reduced weight gain with pH enhancement with ammonia in the LFB (LFBN) group compared to LFB revealed a potential involvement of increased pH in lowering body weight in males (Figure 3A and Supplemental Table S2A). Moreover, a slight reducing effect of pH enhancement on body weights was noted among HFB vs. HFBN and LFB vs. LFBN, but they were not statistically significant (Figure 3A and Supplemental Table S2A). The multiple comparison post hoc test with Tukey correction demonstrated that, HF diet significantly increased final body weight and fat mass regardless of protein source compared to LF fed male mice (LFC; *p* < 0.0001, LFCN; *p* < 0.0001, LFB; *p* = 0.0012, and LFBN; *p* < 0.0001) (Figure 3C and Figure 4A). Additionally, when comparing LF casein groups (LFC, LFCN) to LF beef groups (LFB, LFBN), no significant final body weight or fat mass was observed, indicating that it is not the protein, but dietary fat that is the key contributor for fat over accumulation in the body (Figure 3C). Lean mass was consistent across all the groups except for HFB group in males, which showed slightly higher (statistically significant) lean mass compared to LFC group in male mice (Figure 4D). 

In female mice, all groups demonstrated a similar weight gain pattern throughout the study (Figure 3B & Supplemental Table S2B). Post hoc comparisons indicated significantly higher final body weight in the HFC group compared to the LFC (*p* = 0.0044) and LFCN (*p* = 0.0028) groups (Figure 3D). Surprisingly, none of the beef treated groups (LFB, LFBN, HFB and HFBN) showed significant final body weights compared to the control LFC or LFCN groups in the female mice (Figure 3D). Post hoc comparisons further indicated that the fat mass was significantly higher in HFC group compared to all LF groups (LFC, LFCN, LFB and LFBN) regardless of the protein source (Figure 4B). Interestingly, the pH enhanced HFC group (HFCN) demonstrated significantly less (*p* = 0.0136) fat mass compared to the HFC group, indicating a potential beneficial role of pH enhancement in regulating abdominal fat accumulation (Figure 4B). No statistically significant changes were seen among treatment groups for lean mass for female mice (Figure 4E). 

To identify whether there are sex differences on final body weight, fat mass and lean mass, three-way ANOVA was performed separately for LF, and HF fed mice. As expected, ANOVA results for LF fed mice indicated significant effects of sex on all parameters, including final body weight (F (1, 66) = 213.6, *p* < 0.0001), fat mass (F (1, 59) = 13.33, *p* = 0.0006) and lean mass (F (1, 62) = 491.7, *p* < 0.0001) (Table 3). Other than the sex effect, LF mice indicated a significant effect of protein source on final body weight (F (1, 66) = 16.23, *p* = 0.0001), fat mass (F (1, 59) = 23.29, *p* <0.0001) and lean mass (F (1, 62) = 19.19, *p* < 0.0001) (Table 3) revealing a probable role of beef proteins in increasing muscle mass. HF fed mice also indicated significant effects of sex on all parameters; final body weight (F (1, 65) = 263.4, *p* < 0.0001), fat mass (F (1, 45) = 83.35, *p* < 0.0001) and lean mass (F (1, 51) = 304.2, *p* < 0.0001) (Table 4). As demonstrated in Figure 3E and Figure 4F, final body weight and lean mass were distinctly higher in male groups compared to all female groups, regardless of the diet, protein, and pH enhancement (Figure 3E and Figure 4F). Although the fat mass among male groups was varied between ~2 g to ~15 g, it was still significantly higher compared to fat mass of female mice (Figure 4C). Yet, this ability of beef proteins to increase muscle mass, was either reduced or masked in HF diets as indicated by no significant differences in any of the variables tested, related to body weight (final body weight, fat and lean mass) (Table 4). Interestingly, no significant effect of pH was observed for final body weight, fat mass or lean mass in LF fed male and female mice. However, in HF fed mice, significant effects of pH enhancement were noted on final body weight (F (1, 65) = 4.557, *p* =0.0366) and fat mass (F (1, 45) = 9.442, *p* = 0.0036) indicating that pH enhancement indeed significantly reduced body weight and fat mass compared to non pH enhanced groups (Table 4). These results of pH enhancement or ammonia treatment were more effective in HF diets compared LF diets (Table 3 and Table 4).

### 3.3. Dietary Fat Content and pH Regulate Glucose Clearance, Independent of Sex

**Figure 5:**
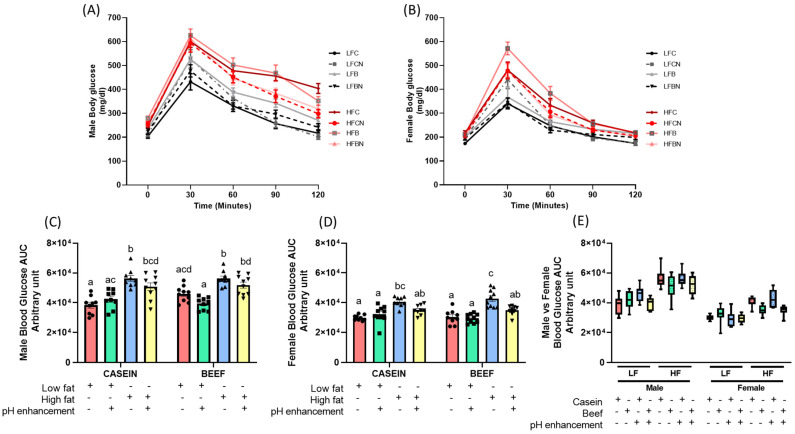
Role of diet, protein source, pH enhancement and sex in glucose clearance in diet induced obese male and female mice. (**A**,**B**) Glucose tolerance test (GTT) in male and female mice. (**C**,**D**) Area under the curve (AUC) for GTT in male and female mice. (**E**) AUC male vs. female. Data is presented as mean ± SEM. LFC, low fat casein; LFCN, low fat casein pH enhanced; LFB, low fat beef; LFBN, low fat beef pH enhanced; HFC, high fat casein; HFCN, high fat casein pH enhanced; HFB, high fat beef; HFBN, high fat beef pH enhanced. Common letters on the error bars indicate no significance (e.g., “a” is significantly different from “b” and “ab” indicates no significance compared to “a” and “b”). *p* < 0.05; *n* = 8–10.

Glucose tolerance was evaluated by performing GTT in both male and female mice. HF diets (all groups HFC, HFCN, HFB, HFBN) significantly increased basal glucose levels in male mice compared to LFC group (Figure 5A and Supplemental Table S3A). In females, only HFC and HFB groups demonstrated significantly higher basal blood glucose levels compared to the LFC group (Figure 5B, and Supplemental Table S3B). No significant effect from protein or pH was observed on basal glucose levels (at week 0) in both male and female mice (Supplemental Table S3A,B). Moreover, in males, LFC groups had significantly (*p* < 0.05) improved glucose tolerance compared to all HF groups throughout the GTT (time points 30, 60, 90 and 120 min) (Supplemental Table S3A). Interestingly, according to the results from the area under the curve, LFB and LFBN groups indicated improved glucose clearance compared to HFB and HFBN groups in male mice, further emphasizing the probable role of dietary fat in metabolic impairments compared to protein source (Figure 5C). Although, the effect of pH enhancement on glucose clearance was not statistically significant from the area under the curve (Figure 5C), post hoc multiple comparisons revealed that over time (from time 0 min to 120 min), pH enhancement significantly improved glucose clearance in males with HF feeding but not with LF (Supplemental Table S3A). Both HFCN and HFBN groups did not show any improvements in glucose clearance at 0-, 30- and 60-min time points compared to HFC group (Supplemental Table S3A). However, the ability to clear glucose was gradually improved at 90-min time point in male HFCN (*p* = 0.0591) and HFBN (*p* = 0.1728) groups compared to HFC groups, while substantially improved glucose clearance was observed in pH enhanced HFCN (*p* = 0.0002) and HFBN (*p* = 0.0057) groups at 120-min time point compared to non pH enhanced HFC groups in male mice (Supplemental Table S3A). In these male mice, glucose clearance was improved by 15–25% in pH enhanced HF groups (HFCN, HFBN) compared to non-pH enhanced HFC and HFB during 90 and 120 time points (Figure 5A). Overall, effects of diet, protein and pH enhancement were markedly greater in male GTT throughout all time points compared to female mice (Figure 5A,B,E). A significant effect of dietary fat content (LF groups vs. HF groups) on glucose clearance was observed in females at 0-, 90- and 120-min time points except for 30 and 60 time points (Figure 5B and Supplemental Table S3B). Like male mice, noticeable effects from the protein source were not observed on glucose clearance in female mice throughout the GTT process (Figure 5B and Supplemental Table S3B). In contrast to the males’ GTT, the effects of pH enhancement on glucose clearance were not observed at the latter part of GTT (time points 90 and 120 min) in female mice (Figure 5B and Supplemental Table S3B). However, when taken together, glucose clearance in Figure 5D (area under the curve), females showed significantly improved glucose clearance in HFBN group compared to the HFB diet confirming the role of pH enhancement with ammonia in improving blood glucose clearance (Figure 5D).

LF feeding regardless of protein source or pH enhancement significantly improved blood glucose clearance in both male and female mice compared to HF fed mice (Table 1 and Table 2) (Figure 5). This was confirmed by three-way ANOVA, which revealed a significant main effect of diet (LF vs. HF) on glucose clearance for both males (F (1, 67) = 77.27, *p* < 0.0001), and females (F (1, 67) = 71.96, *p* < 0.0001) (Table 1 and Table 2), indicating HF diet exacerbates glucose intolerance compared to LF diet. Additionally, pH enhancement significantly improved glucose clearance both in male and female mice, as indicated by the effect of pH on glucose clearance (males (F (1, 67) = 4.896, *p* = 0.0303), and females (F (1, 67) = 8.123, *p* = 0.0058)) (Table 1 and Table 2). When the overall glucose tolerance is considered, pH enhancement in HF fed males (HFCN, HFBN) improved glucose clearance by 8% compared to non-pH enhanced groups (HFC, HFB). A significant diet × pH (D × pH) interaction on glucose clearance was also observed in female mice (Table 2). Post hoc multiple comparisons revealed that both HFC and HFB, regardless of the protein source, had higher glucose intolerance compared to their LF control groups (LFC, LFB), indicated by a higher area under the curve (AUC) in both male and female mice as shown in Figure 5C,D. HF diet, without pH enhancement, demonstrated a strong diabetogenic effect, independent of sex and the protein source, as indicated by significantly higher AUC and blood glucose levels in both male and female mice (Figure 5A–D). Lower dietary fat content was clearly associated with improved glucose clearance in both male and female mice, regardless of the protein source (casein or beef). In addition, pH enhancement significantly improved glucose tolerance in HFB fed (HFBN) female mice compared to the unenhanced controls HFB group (Figure 5D). A similar trend in glucose clearance was observed in female mice, in the HFC vs. HFCN groups (*p* = 0.1042) (Figure 5D). 

Lastly, three-way ANOVA results further confirmed that there is a significant main effect of sex on glucose clearance. Regardless of the diet (LF or HF), female mice showed improved glucose clearance compared to male mice (Table 3 and Table 4, Figure 5E). In LF fed groups along with a considerable effect of sex (F (1, 67) = 101.6, *p* < 0.0001), a significant interaction between pH and protein (pH × P) was noted for glucose clearance. In HF fed mice, significant main effects of sex (F (1, 67) = 146.3, *p* < 0.0001), and pH (F (1, 67) = 20.86, *p* < 0.0001), were observed on glucose clearance further confirming that the effect of pH enhancement is more prevalent in HF diets rather than LF diets (Table 4). However, no significant interactions were indicated among factors for HF fed mice (Table 4). Interestingly, no significant protein effects (casein or beef as the protein source) were observed on glucose clearance in either LF or HF fed male and female mice (Table 3 and Table 4). 

### 3.4. The Effects of Diet and Sex on Serum Metabolic Markers

**Figure 6:**
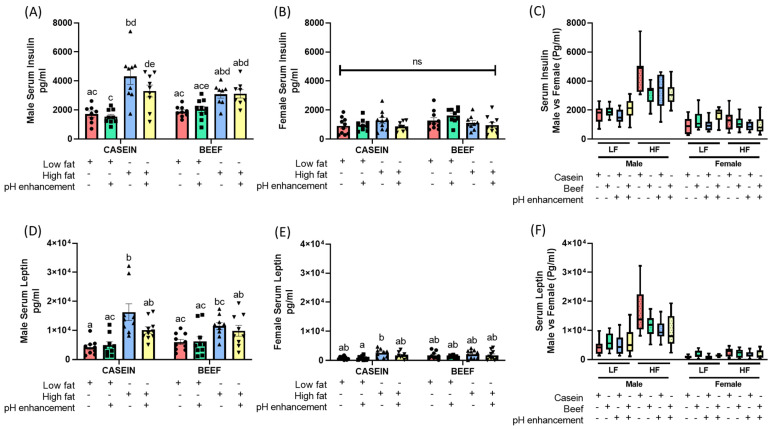
Effects of diet, protein source, pH enhancement and sex on serum metabolic markers in male and female mice. (**A**) Serum insulin levels in male mice. (**B**) Serum insulin levels in female mice. (**C**) Serum insulin levels in male vs. female mice. (**D**) Serum leptin in male mice. (**E**) Serum leptin in female mice. (**F**) Serum leptin levels in male vs. female mice. Data is presented as mean ± SEM. LFC, low fat casein; LFCN, low fat casein pH enhanced; LFB, low fat beef; LFBN, low fat beef pH enhanced; HFC, high fat casein; HFCN, high fat casein pH enhanced; HFB, high fat beef; HFBN, high fat beef pH enhanced. Common letters on the error bars indicate no significance (e.g., “a” is significantly different from “b”, and “ab” indicates no significance compared to “a” and “b”). *p* < 0.05; *n* = 8–10.

High serum insulin and leptin levels are early indicators of insulin resistance and impairments in glucose metabolism/prediabetes [34,35]. As expected, post hoc multiple comparison confirmed that a HF diet with casein as the protein source (HFC) significantly increased serum insulin levels in male mice compared to the LFC group (Figure 6A). However, HF diet with beef as the protein source (HFB) did not have a significantly high serum insulin level compared to control LFC or LFB male groups, indicating an interesting regulatory role of beef in serum insulin levels (Figure 6A). Although it was not statistically significant (*p* = 0.1494), lower serum insulin levels were visible in the HFB group compared to the HFC group in males (Figure 6A). A similar pattern of serum levels was observed for leptin in male mice (Figure 6D). There were no significant differences in serum insulin levels in female mice across all treatment groups (Figure 6B). However, serum leptin level in the HFC group was significantly different compared to LFC groups in females with no statistical differences across other groups (Figure 6E).

Three-way ANOVA revealed significant main effects of diet (LF vs. HF) on both serum insulin (F (1, 62) = 57.37, *p* < 0.0001), and serum leptin level (F (1, 67) = 35.85, *p* < 0.0001) in male mice (Table 1), confirming the involvement of a HF diet in raising serum insulin and leptin levels compared to a LF diet. Neither protein nor pH had any effect on serum insulin levels. However, a significant interaction between protein vs. diet (P × D) was noted in males (Table 1). The main effect of protein, diet or pH did not reach significance for serum insulin levels in female mice (Table 2). However, there were significant interactions between protein vs. diet (P × D) (F (1, 68) = 4.719, *p* = 0.0333) and diet vs. pH (D × pH) (F (1, 68) = 4.088, *p* = 0.0471) (Table 2). Similar to the results in male mice, females also showed a significant effect of diet (LF vs. HF) on serum leptin levels (F (1, 67) = 9.683, *p* = 0.0027) (Table 2). 

To further determine whether there were sex differences on serum insulin and leptin levels, three-way ANOVA was performed separately for LF and HF diet fed mice. As noted for other metabolic parameters, an obvious main effect of sex was observed on serum insulin and leptin levels for both LF and HF fed groups (Table 3 and Table 4). Serum insulin and leptin levels were significantly higher in male mice compared to female mice (Table 3 and Table 4, Figure 6C,F). The interaction effects between sex, protein and pH did not reach significance for the serum insulin level in LF diet mice (Table 3), but there were significant main effects of sex (F (1,66) = 21.83, *p* < 0.0001) and protein (F (1, 66) = 11.01, *p* = 0.0015) on serum insulin levels. Apart from the significant main effects of sex (F (1, 63) = 154.1, *p* < 0.0001) and protein (F (1, 63) = 5.061, *p* = 0.028), a significant effect of pH (F (1, 63) = 5.402, *p* = 0.0234) was also observed in HF diet fed groups for serum insulin levels (Table 4). The levels of serum metabolic markers, including insulin and leptin, were mainly impacted by the diet (LF or HF) and the sex. A strong protein effect (casein vs. beef) was observed on the level of serum markers in males. Unlike glucose tolerance and fat mass results, the effects of pH were barely visible on serum markers across treatment groups.

## 4. Discussion

Previous studies have evaluated the benefits and detrimental health effects of beef as a protein source, yet to best of our knowledge, the literature lacks substantial evidence on whether reported effects are linked to lean meat vs. fat content in the diet, and how altering beef pH affects metabolic outcomes in obesity. Furthermore, no studies have studied metabolic effects of various dietary parameters such as interaction between dietary fat content (LF vs. HF), protein source (beef vs. casein) and pH enhancement (treated with ammonia). Interest in pH enhancement arose from observations that consumption of diets high in fatty meats and low in vegetables may affect metabolic acidosis [5,18,36]. Hence, here we investigated whether diets composed of pH enhanced ground beef or pH enhanced casein would improve metabolic outcomes. Our study has several strengths including novel research on effects of pH enhancement, and including both male and female mice, especially since female data are scarce. Additionally, we studied the overall effects of dietary fat content (LF vs. HF) against protein source and pH enhancement in mice to better understand whether the fat content of the diet or the protein source are the primary regulators of metabolic homeostasis.

The prevalence of obesity has increased to the point that over 42% of American adults are considered obese. High fat and high caloric content of a WD is a major contributing factor to obesity and other NCDs. Potential mechanisms include low grade inflammation and metabolic acidosis [5]. As stated earlier, continuous consumption of foods high in salt, saturated fats and animal proteins/fatty meats could affect acid-base balance in the body causing metabolic acidosis [5,18,36]. A clinical study conducted using renal transplant recipients indicated that high intake of animal protein including meat and fish had significantly lower HCO_3_^−^ serum and serum pH [36]. Moreover, others have shown that Western or acidogenic diet (which is typically high processed foods, salt content and fatty animal proteins) is associated with a rise in the hydrogen ion (H^+^) load in the body, causing impairments in the acid-base balance [22]. Low-grade metabolic acidosis is linked to the progression of several chronic diseases including obesity [37,38]. Thus, reversing or preventing metabolic acidosis could be a potential way to combat disease. Here, we demonstrated the potential role of pH enhancement of dietary proteins (beef or casein) with ammonia treatment, specifically in HF feeding, in reducing fat mass, body weight and improving glucose clearance.

Although there are compensatory processes that function to restore the equilibrium of acid/base balance in the body, continuous consumption of acidogenic diets with increased H^+^ load (chronic metabolic acidosis), could be responsible for altering numerous metabolic pathways, including fat metabolism. Several lines of evidences demonstrated a potential association of weight gain and obesity with lower cellular pH or increased H^+^ load [39]. Our data demonstrated that HF diets, regardless of the protein source, significantly increased body and fat mass. However, this effect was in part reversed by pH enhancement, namely fat mass (in males) and final body weights (in females). These results suggest a probable role of dietary pH or ammonium (used to increased protein pH) in regulating fat accumulation. 

The pH of beef products is generally acidic and may be further exacerbated in the presence of HF, high caloric diets that are also low in vegetables [5,18,26,36]. This cause metabolic impairments partly through inflammation and especially metabolic acidosis. However, studies are lacking or controversial with regard to the effects of beef consumption of cardio metabolic health. Given that beef consumption has started increasing in the US since 2015 according to USDA Food Consumption & Demand report on U.S. Per Capita Availability of Red Meat, Poultry, and Seafood [23], more research is urgently needed to fill this knowledge gap. While research evidence supporting that animal products such as red meat (pork, lamb, beef) may contribute to metabolic acidosis, there is a paucity of studies related to metabolic outcome of changing the pH of meat, particularly in beef, and whether these effects are specific to beef proteins, and/or moderated by fat content in the diet. According to our findings, when the dietary fat content in beef supplemented groups (LFB and LFBN) was reduced, the final body weights and fat mass were significantly decreased compared to the beef group with higher dietary fat content (HFB) in male mice. Similar results were observed in casein protein groups, further confirming that it is the fat content of the diet, which is associated with metabolic imbalances, and not the protein source. While we observed that effects of pH on body weight and weight gain are small but significant, more pronounced effects of pH enhancement may be observed at the cellular and molecular level in target tissues. Therefore, future studies are warranted to understand mechanistic underlying effects of dietary pH in regulating metabolism. 

Impaired glucose metabolism is another potential drawback of increased intracellular acid load. A diet with a high acid load such as that in a typical WD can be responsible for reducing pH of body fluids towards the lower end of the typical pH range (blood and intracellular pH), which could likely be associated with the development of insulin resistance [40]. Early studies conducted by DeFronzo et al., using 16 healthy volunteers revealed that metabolic acidosis due to ammonium chloride administration is responsible for impaired glucose metabolism via reduced tissue insulin sensitivity [41]. Later, another study involving eight chronic renal failure (CRF) patients demonstrated that acidosis indeed contributes to the insulin resistance without affecting the action of insulin [42]. Interestingly, Reaich et al., further proposed that treatment with NaHCO_3_ or increasing pH could result in increased insulin sensitivity [42]. In addition, a positive association between dietary acid load and type 2 diabetes risk was exhibited in a large prospective study conducted using approximately 66,500 women [19]. Corroborating these findings, our research demonstrated that HF diet (represents WD), regardless of protein source (beef or casein), impaired glucose clearance compared to LF beef and casein diets. However, when the pH was enhanced in HF beef (HFB) and HF casein (HFC) diets, blood glucose clearance was improved slowly but significantly in both male and female mice. Lower body weight gain in the pH enhanced HF diets (HFCN, HFBN) in males is likely to be one of the main drivers of the improved glucose metabolism. Therefore, increasing dietary pH of WD either by food processing (treating with ammonium hydroxide) or incorporating foods that reduce net acid load (most fruits and vegetable) could be introduced to reduce the possible risk of metabolic impairments linked to chronic diseases including obesity and diabetes.

With regard to obesity, evidence linking red meat consumption to diabetes risk is debatable. Van Dam et al. in early 2000 revealed that regular consumption of processed meats (sausages and bacon with nitrites) may increase the risk of T2D, but consumption of beef, lamb, or pork as a main dish or a mixed dish was not significantly associated with T2D risk [43]. Additionally, their study revealed that the fat intake (total and saturated fat) may have a higher risk of T2D [43]. In fact, as revealed by a recent study, the energy intake was significantly greater in adults who consumed ultra-processed diets compared to consumers of unprocessed diet [7]. Weight gain was highly correlated with increased energy intake in ultra-processed diet group increasing the risk of obesity and associated diseases including T2D [7]. On the contrary, an updated meta-analysis using US adults by Panet et al. [44] and two other recent studies (9 year prospective cohort study on Chinees adults and a meta-analysis) [45,46], indicated that although the relative risk for T2D is less compared to processed meat, unprocessed red meat consumption still has a substantial risk of getting T2D [44,45,46]. Nevertheless, it is still unclear whether the later risk of T2D is solely related to meat consumption, or it is partly due to other dietary factors such as fat content, salt and total acid load coming from other foods consumed along with unprocessed meat that could contribute to T2D risk. For example, a plethora of evidence supports that WD rich in salts [47], fried food [48,49,50,51] and non-meat related fats [52,53] impair glucose and insulin metabolism, causing chronic diseases including T2D, obesity and CVDs. Supporting this argument, our data demonstrated that HF diets regardless of the protein source (beef or casein) caused impairments in glucose clearance compared to LF beef or LF casein groups, further emphasizing a potentially stronger role of dietary fat over other factors such as protein source/meat in metabolic impairments. 

According to Gannonet al., a high-protein diet (containing beef), lowered blood glucose in individuals with T2D compared to a prototypical healthy (control) diet [54]. Possibly, the insulinotropic effect of dietary protein including beef, towards increasing insulin secretion, contributes to enhanced glucose clearance from the blood [55]. Moreover, higher release of insulin is associated with improved glucose clearance [56]. Yet, increased serum insulin levels, with reduced glucose clearance observed in HF casein group, may be an early indicator of insulin resistance. Additionally, substantially reduced serum insulin and leptin levels observed in LF groups, regardless of protein (beef or casein) or pH enhancement, further confirms that dietary fat content may play a significant part in metabolic dysregulation. On the other hand, female mice did not show any significant differences in serum insulin or leptin levels across treatments. This could be due to B6 females having better mechanisms to cope with HF diet and are less susceptible to diet-induced metabolic impairments as shown elsewhere [57,58]. 

Apart from the detrimental effects associated with overconsumption of dietary fat (total and saturated fat), excessive release of acids (H^+^ ions) into the bloodstream and intracellular fluids may influence various metabolic imbalances including impairments in fat metabolism, insulin resistance and increased mineral excretion. These imbalances may partly be responsible for increased risk of non-communicable diseases such as obesity, T2D and CVDs. Thus, controlling or limiting the excessive release of acids into body fluids would be an ideal way to combat diseases linked to metabolic acidosis. Our approach of enhancing pH of ground beef and casein to reduce the acid load, showed promising improvements in terms of fat and glucose metabolism. Despite enhancing the pH of dietary proteins to pH ≈ 9 with ammonium hydroxide, the final pH of the diet (once proteins (beef or casein) are mixed into the diets) was around 7.5 to 8 (Supplemental Table S1). Therefore, it would be interesting to investigate if increasing pH of the whole diet would be more beneficial. Additionally, it is important to dissect the molecular mechanisms via which the changes of dietary pH would regulate metabolic pathways. Future studies are required to further evaluate the role of pH in metabolism. The study has a few limitations, including not varying sources of fat. We adjusted our dietary fat levels with beef tallow, and it is possible that other fat sources such as lard or fish oil may have different effects. We also encountered some technical challenges during this study, as certain instruments unexpectedly stopped operating while taking measurements (reducing the sample numbers for some metabolic measurements to 4 or 5 in some experiments). 

## 5. Conclusions

Overall, these studies demonstrate for the first-time improvements in markers of metabolic health, especially body weight, body fat and glucose clearance by increasing pH of dietary proteins (beef, casein). The main effects and interactions observed in diet, pH and protein source further indicate that it is the amount of fat that contributes to metabolic dysfunctions of the high fat diet, not the source of protein. Lastly, we identified sex differences across all variables tested (glucose clearance, final body weight, fat mass, lean mass, serum leptin and insulin levels) regardless of dietary fat content (LF or HF), further confirming the sex-dependent impact on metabolism regardless of the diet, protein source or the whether the diet pH is enhanced or not. Whether reformulation of WD or replacing it with a diet rich in lean meat and less acid forming food could eliminate deleterious effects associated with metabolic acidosis is unclear. Yet, limiting dietary fat content and increasing lean protein intake along with enhanced dietary pH may be an effective strategy for preventing metabolic alterations linked with obesity and related chronic diseases including T2D. However, these findings merit further translational research into clinical studies before making specific dietary recommendations.

## Figures and Tables

**Table 1:** Effects of protein, diet, and pH on body weight and body composition, glucose tolerance and serum markers in B6 male mice. *p* and F values of three-way ANOVA to evaluate main effect and interactions.

		Main Effects			Interactions			
Variable	Statistic	Protein (P)	Diet (D)	pH (H)	P × D	P × H	D × H	P × D × H
*Final Body Weight*	*p*	0.3106	**<0.0001**	0.1222	0.0754	0.4275	0.339	0.3473
	F (1, 67)	1.044	90.27	2.45	3.261	0.6374	0.9274	0.3473
*Fat Mass*	*p*	**0.0058**	**<0.0001**	**0.0312**	0.1929	0.3824	0.351	0.5234
	F (1, 39)	8.517	55.64	4.996	1.755	0.7806	0.8911	0.4145
*Lean Mass*	*p*	**0.0107**	**0.0397**	0.1315	0.1045	0.3682	**0.0287**	0.2143
	F (1, 41)	7.151	4.512	2.368	2.756	0.8279	5.14	1.591
*Glucose Tolerance*	*p*	0.3203	**<0.0001**	**0.0303**	0.5068	0.1034	0.1887	**0.0427**
	F (1, 67)	1.003	77.27	4.896	0.4454	2.725	1.764	4.27
*Serum Insulin*	*p*	0.4076	**<0.0001**	0.2626	**0.0198**	0.1234	0.2888	0.4227
	F (1, 62)	0.6951	57.37	1.278	5.721	2.439	1.145	0.6515
*Serum Leptin*	*p*	0.6695	**<0.0001**	0.1244	0.0678	0.3673	0.0544	0.257
	F (1, 67)	0.1838	35.85	2.422	3.446	0.824	3.832	1.307

P, Protein (Casein or beef); D, Diet (LF or HF); H, pH (pH not enhanced or enhanced), × indicates interactions, data are expressed as mean ± SEM, values bolded to highlight significance.

**Table 2:** Effects of protein, diet, and pH on body weight and body composition, glucose tolerance and serum markers in B6 female mice. P and F values of three-way ANOVA to evaluate main effect and interactions.

		Main Effects			Interactions			
Variable	Statistic	Protein (P)	Diet (D)	pH (H)	P × D	P × H	D × H	P × D × H
*Final Body Weight*	*p*	0.6346	**0.0362**	**0.0296**	**0.0101**	0.3357	0.4105	**0.0412**
	F (1, 64)	0.228	4.578	4.952	7.027	0.9408	0.6862	4.342
*Fat Mass*	*p*	0.709	**0.0003**	0.0574	**0.0152**	0.0767	**0.0286**	0.1438
	F (1, 65)	0.1405	14.96	3.743	6.219	3.236	5.014	2.19
*Lean Mass*	*p*	0.4621	0.2344	0.913	0.1049	0.9447	0.4794	0.9316
	F (1, 72)	0.5466	1.438	0.01202	2.698	0.00484	0.5055	0.007409
*Glucose Tolerance*	*p*	0.6806	**<0.0001**	**0.0058**	0.1644	0.2628	**0.0001**	0.9436
	F (1, 67)	0.1709	71.96	8.123	1.976	1.275	17.01	0.005036
*Serum Insulin*	*p*	0.0578	0.2596	0.7709	**0.0333**	0.2827	**0.0471**	0.9625
	F (1, 68)	3.725	1.292	0.08547	4.719	1.173	4.088	0.002224
*Serum Leptin*	*p*	0.3825	**0.0027**	0.1099	0.098	0.9244	0.5423	0.4474
	F (1, 67)	0.7728	9.683	2.625	2.816	0.009082	0.3751	0.5841

P, Protein (Casein or beef); D, Diet (LF or HF); H, pH (pH not enhanced or enhanced), × indicates interactions, data are expressed as mean ± SEM, values bolded to highlight significance.

**Table 3:** Effects of sex, pH, and protein in on body weight and body composition, glucose tolerance and serum markers in low fat (LF) diet-fed B6 mice. *p* and F values of three-way ANOVA to evaluate main effect and interactions.

		Main Effects			Interactions			
Variable	Statistic	Sex (S)	pH (H)	Protein (P)	S × H	S × P	H × P	S × H × P
*Final Body Weight*	*p*	**<0.0001**	0.2188	**0.0001**	0.8231	0.4582	0.0436	0.3772
	F (1, 66)	213.6	1.541	16.23	0.05036	0.5569	4.235	0.7905
*Fat Mass*	*p*	**0.0006**	0.3307	**<0.0001**	0.2224	**0.0173**	0.9419	0.7368
	F (1, 59)	13.33	0.9619	23.29	1.521	5.999	0.005365	0.1141
*Lean Mass*	*p*	**<0.0001**	0.9177	**<0.0001**	0.3439	0.0603	0.7381	0.8619
	F (1, 62)	491.7	0.01078	19.19	0.9097	3.662	0.1128	0.0305
*Glucose Tolerance*	*p*	**<0.0001**	0.995	0.7938	0.2754	0.0727	**0.0071**	0.0659
	F (1, 67)	101.6	0.00003902	0.06887	1.21	3.326	7.716	3.495
*Serum Insulin*	*p*	**<0.0001**	0.4374	**0.0015**	0.3791	0.5267	0.2368	0.917
	F (1, 66)	21.83	0.6106	11.01	0.7842	0.405	1.425	0.01095
*Serum Leptin*	*p*	**<0.0001**	0.8863	0.0756	0.5718	0.4699	0.7277	0.9462
	F (1, 67)	43.3	0.02059	3.256	0.3229	0.5282	0.1223	0.00459

P, Protein (Casein or beef); S, Sex (Male or Female); H, pH (pH not enhanced or enhanced), × indicates interactions, data are expressed as mean ± SEM, values bolded to highlight significance.

**Table 4:** Effects of sex, pH, and protein on body weight and body composition, glucose tolerance and serum markers in high fat (HF) diet-fed B6 mice. *p* and F values of three-way ANOVA to evaluate main effect and interactions.

		Main Effects			Interactions			
Variable	Statistic	Sex (S)	pH (H)	Protein (P)	S × H	S × P	H × P	S × H × P
*Final Body Weight*	*p*	**<0.0001**	**0.0366**	0.307	0.7619	0.7856	0.3275	0.4019
	F (1, 65)	263.4	4.557	1.06	0.09261	0.07461	0.9734	0.7119
*Fat Mass*	*p*	**<0.0001**	**0.0036**	0.7379	0.4955	0.1287	0.9732	0.0723
	F (1, 45)	83.35	9.442	0.1134	0.4721	2.396	0.001142	3.386
*Lean Mass*	*p*	**<0.0001**	0.1127	0.8603	**0.0482**	0.4317	0.3017	0.3069
	F (1, 51)	304.2	2.605	0.03127	4.097	0.6281	1.089	1.065
*Glucose Tolerance*	*p*	**<0.0001**	**<0.0001**	0.5796	0.4963	0.8495	0.8235	0.499
	F (1, 67)	146.3	20.86	0.3099	0.4678	0.03628	0.05016	0.462
*Serum Insulin*	*p*	**<0.0001**	**0.0234**	**0.028**	0.3719	**0.0397**	**0.0473**	0.1708
	F (1, 63)	154.1	5.402	5.061	0.8088	4.412	4.093	1.92
*Serum Leptin*	*p*	**<0.0001**	**0.0216**	0.1547	0.0874	0.2274	0.1942	0.2867
	F (1, 67)	108.6	5.532	2.072	3.009	1.484	1.72	1.153

P, Protein (Casein or beef); S, Sex (Male or Female); H, pH (pH not enhanced or enhanced), × indicates interactions, data are expressed as mean ± SEM, values bolded to highlight significance.

## Data Availability

Not applicable.

## Supplementary Materials

The following supporting information can be downloaded at: https://www.mdpi.com/article/10.3390/nu14132583/s1, Table S1: Diet Composition and diet pH; Table S2A: The effects of dietary fat, protein source and pH enhancement in weekly body weight in male mice; Table S2B: The effects of dietary fat, protein source and pH enhancement in weekly body weight in female mice; Table S3A: Male glucose tolerance test results (Time point 0 to 120 min); Table S3B: Female glucose tolerance test results (Time point 0 to 120 min).
