# Supplementary material for: Dietary pH Enhancement Improves Metabolic Outcomes in Diet-Induced Obese Male and Female Mice: Effects of Beef vs. Casein Proteins
Source: Nutrients. 2022 Jun 22;14(13):2583. doi: 10.3390/nu14132583 (PMC9268221; doi:10.3390/nu14132583)
Supplement: Supplementary file 1 [file nutrients-14-02583-s001.zip › Supplementary Table S1_Diet Composition and Diet pH.pdf]

Diets	LF Casein	LF Casein pH-Enhanced	LF Beef	LF lean Beef pH-Enhanced	HF Casein	HF Casein pH-Enhanced	HF Beef	HF Beef pH-Enhanced
**Ingredients**								
Casein	200	0	0	0	200	0	0	0
Freeze Dried Casein, "pH-Enhanced" (RDI Lot #s 34318 OR 35568)	0	186.98	0	0	0	186.98	0	0
Beef, Cooked, Freeze Dried, pH-Enhanced (RDI Lot # 35569)	0	0	0	0	0	0	0	312.17
Beef, Cooked, Freeze Dried, non-pH-Enhanced (RDI Lot # 35570	0	0	0	0	0	0	265.14	0
Freeze Dried 98% Lean Beef; pH-Enhanced (RDI Lot # 35604)	0	0	0	201.86	0	0	0	0
Freeze Dried 98% Lean Beef; non-pH-Enhanced (RDI Lot # 35603)	0	0	202.88	0	0	0	0	0
L-Cystine	3	3	3	3	3	3	3	3
Corn Starch	452.2	452.2	451.2	451.2	72.8	72.8	71.46	71.23
Maltodextrin 10	75	75	75	75	100	100	100	100
Sucrose	175.21	175.21	175.21	175.21	175.21	175.21	175.21	175.21
Cellulose	50	50	47.8	48.2	50	50	48.14	47.5
Soybean Oil	25	25	25	25	25	25	25	25
Beef Fat, Bunge	20	20	1	1.1	177.5	177.5	100.358	55.03
Mineral Mix S10026A (No Ca, P, K, Na, Cl)	5	5	5	5	5	5	5	5
Dicalcium Phosphate	13	13	13	13	13	13	13	13
Calcium Carbonate	5.5	5.5	5.5	5.5	5.5	5.5	5.5	5.5
Potassium Citrate, 1 H2O	16.5	16.5	16.5	16.5	16.5	16.5	16.5	16.5
Sodium Chloride	2.546	2.546	1.753	1.74	2.546	2.546	1.157	1.263
Vitamin Mix V10001	10	10	10	10	10	10	10	10
Choline Bitartrate	2	2	2	2	2	2	2	2
Cholesterol	0.6	0.6	0.24	0.25	0.49	0.49	0.05	0.02
Yellow Dye #5, FD&C	0	0.025	0.04	0.04	0	0.05	0.025	0
Red Dye #40, FD&C	0.05	0	0.01	0	0	0	0.025	0.025
Blue Dye #1, FD&C	0	0.025	0	0.01	0.05	0	0	0.025
Total	1055.606	1042.586	1035.133	1034.61	858.596	845.576	841.565	842.473
**gm**								
Protein	179	178.6	179	179	179	178.6	179	179
Carbohydrate	712.4	712.4	712.4	712.4	358	358	358	358
Fat	47.4	47.4	47.4	47.4	204.9	204.9	204.9	204.9
Fiber	50	50	50	50	50	50	50	50
Cholesterol	0.66	0.65	0.66	0.66	0.66	0.66	0.66	0.66

**gm %**								
Protein	17	17.1	17.3	17.3	20.8	21.1	21.3	21.2
Carbohydrate	67.5	68.3	68.8	68.9	41.7	42.3	42.5	42.5
Fat	4.5	4.5	4.6	4.6	23.9	24.2	24.3	24.3
Fiber	4.7	4.8	48	4.8	5.8	5.9	5.9	5.9
Cholesterol	0.062	0.063	0.064	0.064	0.08	0.08	0.08	0.08
**kcals**								
Protein	716	714.5	716	716	716	714.5	716	716
Carbohydrate	2849.6	2849.6	2849.7	2849.7	1432	1432	1432	1432
Fat	426.6	426.6	426.8	426.7	1844.1	1844.1	1844.1	1844.1
Total	3992.2	3990.7	3992.5	3992.4	3992.1	3990.6	3992.1	3992.1
**kcals %**								
Protein	18	18	18	18	18	18	18	18
Carbohydrate	71	71	71	71	36	36	36	36
Fat	11	11	11	11	46	46	46	46
Total	100	100	100	100	100	100	100	100
kcal/gm	3.8	3.8	3.9	3.9	4.6	4.7	4.7	4.7
**Diet pH**	6.637 ± 0.059 a	7.73 ± 0.072 b	7.257 ± 0.003 c	7.557 ± 0.014 bc	6.263± 0.024 d	7.895 ± 0.068 b	6.873 ± 0.037 e	7.375 ± 0.048 c

Values are means ± SEMs, *n* = 6. Means without a common superscript letter are significantly different.

Supplemental Table S1: Diet Composition and diet pH.
