# Supplementary material for: Dietary pH Enhancement Improves Metabolic Outcomes in Diet-Induced Obese Male and Female Mice: Effects of Beef vs. Casein Proteins
Source: Nutrients. 2022 Jun 22;14(13):2583. doi: 10.3390/nu14132583 (PMC9268221; doi:10.3390/nu14132583)
Supplement: Supplementary file 1 [file nutrients-14-02583-s001.zip › Supplementary Table S2A_Male Weekly Body Weights.pdf]

Supplementary Table S2A: The effects of dietary fat, protein source and pH enhancement in weekly body weight in male mice.
Legends: LFC, low fat casein; LFCN, low fat casein pH enhanced; LFB, low fat beef; LFBN, low fat beef pH enhanced; HFC, high fat casein; HFCN, high fat casein pH enhanced; HFB, high fat beef; HFBN, high fat beef pH enhanced.

Week 0:

Tukey's multiple comparisons test	Mean Diff.	95.00% CI of diff.	Significant?	Summary	Adjusted P Value
LFC vs. LFCN	-0,18	-4.122 to 3.762	No	ns	>0.9999
LFC vs. LFB	-2,11	-5.524 to 1.304	No	ns	0,5187
LFC vs. LFBN	-0,09	-3.504 to 3.324	No	ns	>0.9999
LFC vs. HFC	-1,02	-4.962 to 2.922	No	ns	0,9911
LFC vs. HFCN	-1,36	-5.302 to 2.582	No	ns	0,955
LFC vs. HFB	0,1	-3.842 to 4.042	No	ns	>0.9999
LFC vs. HFBN	-1,11	-4.524 to 2.304	No	ns	0,9671
LFCN vs. LFB	-1,93	-5.344 to 1.484	No	ns	0,6279
LFCN vs. LFBN	0,09	-3.324 to 3.504	No	ns	>0.9999
LFCN vs. HFC	-0,84	-4.782 to 3.102	No	ns	0,9973
LFCN vs. HFCN	-1,18	-5.122 to 2.762	No	ns	0,9792
LFCN vs. HFB	0,28	-3.662 to 4.222	No	ns	>0.9999
LFCN vs. HFBN	-0,93	-4.344 to 2.484	No	ns	0,9879
LFB vs. LFBN	2,02	-0.7677 to 4.808	No	ns	0,3163
LFB vs. HFC	1,09	-2.324 to 4.504	No	ns	0,9702
LFB vs. HFCN	0,75	-2.664 to 4.164	No	ns	0,9967
LFB vs. HFB	2,21	-1.204 to 5.624	No	ns	0,4592
LFB vs. HFBN	1	-1.788 to 3.788	No	ns	0,9449
LFBN vs. HFC	-0,93	-4.344 to 2.484	No	ns	0,9879
LFBN vs. HFCN	-1,27	-4.684 to 2.144	No	ns	0,9338
LFBN vs. HFB	0,19	-3.224 to 3.604	No	ns	>0.9999
LFBN vs. HFBN	-1,02	-3.808 to 1.768	No	ns	0,939
HFC vs. HFCN	-0,34	-4.282 to 3.602	No	ns	>0.9999
HFC vs. HFB	1,12	-2.822 to 5.062	No	ns	0,9845
HFC vs. HFBN	-0,09	-3.504 to 3.324	No	ns	>0.9999
HFCN vs. HFB	1,46	-2.482 to 5.402	No	ns	0,9352
HFCN vs. HFBN	0,25	-3.164 to 3.664	No	ns	>0.9999
HFB vs. HFBN	-1,21	-4.624 to 2.204	No	ns	0,9482

Week 1:

Tukey's multiple comparisons test	Mean Diff.	95.00% CI of diff.	Significant?	Summary	Adjusted P Value
LFC vs. LFCN	-0,89	-4.161 to 2.381	No	ns	0,9894
LFC vs. LFB	-0,7	-3.971 to 2.571	No	ns	0,9976
LFC vs. LFBN	0,61	-2.661 to 3.881	No	ns	0,999
LFC vs. HFC	-2,04	-5.311 to 1.231	No	ns	0,5233
LFC vs. HFCN	-2,19	-5.461 to 1.081	No	ns	0,4303
LFC vs. HFB	-1,28	-4.551 to 1.991	No	ns	0,9224
LFC vs. HFBN	-0,5	-3.771 to 2.771	No	ns	0,9997
LFCN vs. LFB	0,19	-2.894 to 3.274	No	ns	>0.9999
LFCN vs. LFBN	1,5	-1.584 to 4.584	No	ns	0,7943
LFCN vs. HFC	-1,15	-4.234 to 1.934	No	ns	0,9391
LFCN vs. HFCN	-1,3	-4.384 to 1.784	No	ns	0,8894
LFCN vs. HFB	-0,39	-3.474 to 2.694	No	ns	>0.9999
LFCN vs. HFBN	0,39	-2.694 to 3.474	No	ns	>0.9999
LFB vs. LFBN	1,31	-1.774 to 4.394	No	ns	0,8854
LFB vs. HFC	-1,34	-4.424 to 1.744	No	ns	0,8729
LFB vs. HFCN	-1,49	-4.574 to 1.594	No	ns	0,7998
LFB vs. HFB	-0,58	-3.664 to 2.504	No	ns	0,9989
LFB vs. HFBN	0,2	-2.884 to 3.284	No	ns	>0.9999
LFBN vs. HFC	-2,65	-5.734 to 0.4341	No	ns	0,1447
LFBN vs. HFCN	-2,8	-5.884 to 0.2841	No	ns	0,1025
LFBN vs. HFB	-1,89	-4.974 to 1.194	No	ns	0,5458
LFBN vs. HFBN	-1,11	-4.194 to 1.974	No	ns	0,9492
HFC vs. HFCN	-0,15	-3.234 to 2.934	No	ns	>0.9999
HFC vs. HFB	0,76	-2.324 to 3.844	No	ns	0,9941
HFC vs. HFBN	1,54	-1.544 to 4.624	No	ns	0,7718
HFCN vs. HFB	0,91	-2.174 to 3.994	No	ns	0,9829
HFCN vs. HFBN	1,69	-1.394 to 4.774	No	ns	0,6795
HFB vs. HFBN	0,78	-2.304 to 3.864	No	ns	0,9931

Week 2:

Tukey's multiple comparisons test	Mean Diff.	95.00% CI of diff.	Significant?	Summary	Adjusted P Value
LFC vs. LFCN	-0,9775	-4.077 to 2.122	No	ns	0,9751
LFC vs. LFB	-1,378	-4.477 to 1.722	No	ns	0,8594
LFC vs. LFBN	-0,2775	-3.377 to 2.822	No	ns	>0.9999
LFC vs. HFC	-2,538	-5.637 to 0.5622	No	ns	0,1893
LFC vs. HFCN	-2,738	-5.837 to 0.3622	No	ns	0,1225
LFC vs. HFB	-2,648	-5.747 to 0.4522	No	ns	0,1498
LFC vs. HFBN	-1,728	-4.827 to 1.372	No	ns	0,6606
LFCN vs. LFB	-0,4	-3.322 to 2.522	No	ns	0,9999
LFCN vs. LFBN	0,7	-2.222 to 3.622	No	ns	0,9951
LFCN vs. HFC	-1,56	-4.482 to 1.362	No	ns	0,7074
LFCN vs. HFCN	-1,76	-4.682 to 1.162	No	ns	0,5678
LFCN vs. HFB	-1,67	-4.592 to 1.252	No	ns	0,6318
LFCN vs. HFBN	-0,75	-3.672 to 2.172	No	ns	0,9925
LFB vs. LFBN	1,1	-1.822 to 4.022	No	ns	0,9361
LFB vs. HFC	-1,16	-4.082 to 1.762	No	ns	0,9168
LFB vs. HFCN	-1,36	-4.282 to 1.562	No	ns	0,8285
LFB vs. HFB	-1,27	-4.192 to 1.652	No	ns	0,8728
LFB vs. HFBN	-0,35	-3.272 to 2.572	No	ns	>0.9999
LFBN vs. HFC	-2,26	-5.182 to 0.6624	No	ns	0,25
LFBN vs. HFCN	-2,46	-5.382 to 0.4624	No	ns	0,1628
LFBN vs. HFB	-2,37	-5.292 to 0.5524	No	ns	0,1988
LFBN vs. HFBN	-1,45	-4.372 to 1.472	No	ns	0,7774
HFC vs. HFCN	-0,2	-3.122 to 2.722	No	ns	>0.9999
HFC vs. HFB	-0,11	-3.032 to 2.812	No	ns	>0.9999
HFC vs. HFBN	0,81	-2.112 to 3.732	No	ns	0,9881
HFCN vs. HFB	0,09	-2.832 to 3.012	No	ns	>0.9999
HFCN vs. HFBN	1,01	-1.912 to 3.932	No	ns	0,959
HFB vs. HFBN	0,92	-2.002 to 3.842	No	ns	0,9753

Week 3:

Tukey's multiple comparisons test	Mean Diff.	95.00% CI of diff.	Significant?	Summary	Adjusted P Value
LFC vs. LFCN	-1,163	-4.328 to 2.003	No	ns	0,9436
LFC vs. LFB	-1,943	-5.108 to 1.223	No	ns	0,5441
LFC vs. LFBN	-0,4125	-3.578 to 2.753	No	ns	>0.9999
LFC vs. HFC	-3,283	-6.448 to -0.1169	Yes	*	0,0367
LFC vs. HFCN	-4,313	-7.478 to -1.147	Yes	**	0,0016
LFC vs. HFB	-3,393	-6.558 to -0.2269	Yes	*	0,0271
LFC vs. HFBN	-2,793	-5.958 to 0.3731	No	ns	0,1234
LFCN vs. LFB	-0,78	-3.765 to 2.205	No	ns	0,9916
LFCN vs. LFBN	0,75	-2.235 to 3.735	No	ns	0,9934
LFCN vs. HFC	-2,12	-5.105 to 0.8646	No	ns	0,3532
LFCN vs. HFCN	-3,15	-6.135 to -0.1654	Yes	*	0,0313
LFCN vs. HFB	-2,23	-5.215 to 0.7546	No	ns	0,2903
LFCN vs. HFBN	-1,63	-4.615 to 1.355	No	ns	0,6831
LFB vs. LFBN	1,53	-1.455 to 4.515	No	ns	0,7477
LFB vs. HFC	-1,34	-4.325 to 1.645	No	ns	0,853
LFB vs. HFCN	-2,37	-5.355 to 0.6146	No	ns	0,2208
LFB vs. HFB	-1,45	-4.435 to 1.535	No	ns	0,7952
LFB vs. HFBN	-0,85	-3.835 to 2.135	No	ns	0,9861
LFBN vs. HFC	-2,87	-5.855 to 0.1146	No	ns	0,0681
LFBN vs. HFCN	-3,9	-6.885 to -0.9154	Yes	**	0,0028
LFBN vs. HFB	-2,98	-5.965 to 0.004589	No	ns	0,0506
LFBN vs. HFBN	-2,38	-5.365 to 0.6046	No	ns	0,2163
HFC vs. HFCN	-1,03	-4.015 to 1.955	No	ns	0,9593
HFC vs. HFB	-0,11	-3.095 to 2.875	No	ns	>0.9999
HFC vs. HFBN	0,49	-2.495 to 3.475	No	ns	0,9996
HFCN vs. HFB	0,92	-2.065 to 3.905	No	ns	0,9781
HFCN vs. HFBN	1,52	-1.465 to 4.505	No	ns	0,7539
HFB vs. HFBN	0,6	-2.385 to 3.585	No	ns	0,9984

Week 4:

Tukey's multiple comparisons test	Mean Diff.	95.00% CI of diff.	Significant?	Summary	Adjusted P Value
LFC vs. LFCN	-0,8256	-3.634 to 1.983	No	ns	0,9833
LFC vs. LFB	-1,826	-4.634 to 0.9832	No	ns	0,4697
LFC vs. LFBN	-0,3356	-3.144 to 2.473	No	ns	>0.9999
LFC vs. HFC	-3,516	-6.324 to -0.7068	Yes	**	0,0049
LFC vs. HFCN	-3,356	-6.164 to -0.5468	Yes	**	0,0086
LFC vs. HFB	-3,516	-6.324 to -0.7068	Yes	**	0,0049
LFC vs. HFBN	-2,806	-5.614 to 0.003165	No	ns	0,0505
LFCN vs. LFB	-1	-3.734 to 1.734	No	ns	0,9449
LFCN vs. LFBN	0,49	-2.244 to 3.224	No	ns	0,9992
LFCN vs. HFC	-2,69	-5.424 to 0.04381	No	ns	0,057
LFCN vs. HFCN	-2,53	-5.264 to 0.2038	No	ns	0,09
LFCN vs. HFB	-2,69	-5.424 to 0.04381	No	ns	0,057
LFCN vs. HFBN	-1,98	-4.714 to 0.7538	No	ns	0,3289
LFB vs. LFBN	1,49	-1.244 to 4.224	No	ns	0,6858
LFB vs. HFC	-1,69	-4.424 to 1.044	No	ns	0,5351
LFB vs. HFCN	-1,53	-4.264 to 1.204	No	ns	0,6564
LFB vs. HFB	-1,69	-4.424 to 1.044	No	ns	0,5351
LFB vs. HFBN	-0,98	-3.714 to 1.754	No	ns	0,9504
LFBN vs. HFC	-3,18	-5.914 to -0.4462	Yes	*	0,0117
LFBN vs. HFCN	-3,02	-5.754 to -0.2862	Yes	*	0,0202
LFBN vs. HFB	-3,18	-5.914 to -0.4462	Yes	*	0,0117
LFBN vs. HFBN	-2,47	-5.204 to 0.2638	No	ns	0,1059
HFC vs. HFCN	0,16	-2.574 to 2.894	No	ns	>0.9999
HFC vs. HFB	0	-2.734 to 2.734	No	ns	>0.9999
HFC vs. HFBN	0,71	-2.024 to 3.444	No	ns	0,992
HFCN vs. HFB	-0,16	-2.894 to 2.574	No	ns	>0.9999
HFCN vs. HFBN	0,55	-2.184 to 3.284	No	ns	0,9984
HFB vs. HFBN	0,71	-2.024 to 3.444	No	ns	0,992

Week 5:

Tukey's multiple comparisons test	Mean Diff.	95.00% CI of diff.	Significant?	Summary	Adjusted P Value
LFC vs. LFCN	-1,481	-4.699 to 1.737	No	ns	0,8367
LFC vs. LFB	-2,601	-5.819 to 0.6168	No	ns	0,2023
LFC vs. LFBN	-1,101	-4.319 to 2.117	No	ns	0,9612
LFC vs. HFC	-4,121	-7.339 to -0.9032	Yes	**	0,0037
LFC vs. HFCN	-4,181	-7.399 to -0.9632	Yes	**	0,003
LFC vs. HFB	-4,411	-7.629 to -1.193	Yes	**	0,0014
LFC vs. HFBN	-3,431	-6.649 to -0.2132	Yes	*	0,0285
LFCN vs. LFB	-1,12	-4.252 to 2.012	No	ns	0,951
LFCN vs. LFBN	0,38	-2.752 to 3.512	No	ns	>0.9999
LFCN vs. HFC	-2,64	-5.772 to 0.4921	No	ns	0,1618
LFCN vs. HFCN	-2,7	-5.832 to 0.4321	No	ns	0,1421
LFCN vs. HFB	-2,93	-6.062 to 0.2021	No	ns	0,0834
LFCN vs. HFBN	-1,95	-5.082 to 1.182	No	ns	0,5259
LFB vs. LFBN	1,5	-1.632 to 4.632	No	ns	0,8072
LFB vs. HFC	-1,52	-4.652 to 1.612	No	ns	0,7965
LFB vs. HFCN	-1,58	-4.712 to 1.552	No	ns	0,7631
LFB vs. HFB	-1,81	-4.942 to 1.322	No	ns	0,619
LFB vs. HFBN	-0,83	-3.962 to 2.302	No	ns	0,9909
LFBN vs. HFC	-3,02	-6.152 to 0.1121	No	ns	0,0667
LFBN vs. HFCN	-3,08	-6.212 to 0.05213	No	ns	0,0573
LFBN vs. HFB	-3,31	-6.442 to -0.1779	Yes	*	0,0309
LFBN vs. HFBN	-2,33	-5.462 to 0.8021	No	ns	0,2959
HFC vs. HFCN	-0,06	-3.192 to 3.072	No	ns	>0.9999
HFC vs. HFB	-0,29	-3.422 to 2.842	No	ns	>0.9999
HFC vs. HFBN	0,69	-2.442 to 3.822	No	ns	0,9971
HFCN vs. HFB	-0,23	-3.362 to 2.902	No	ns	>0.9999
HFCN vs. HFBN	0,75	-2.382 to 3.882	No	ns	0,9951
HFB vs. HFBN	0,98	-2.152 to 4.112	No	ns	0,9762

Week 6:

Tukey's multiple comparisons test	Mean Diff.	95.00% CI of diff.	Significant?	Summary	Adjusted P Value
LFC vs. LFCN	-1,018	-4.370 to 2.335	No	ns	0,98
LFC vs. LFB	-2,848	-6.200 to 0.5046	No	ns	0,1548
LFC vs. LFBN	-1,718	-5.070 to 1.635	No	ns	0,7485
LFC vs. HFC	-4,908	-8.260 to -1.555	Yes	***	0,0005
LFC vs. HFCN	-5,278	-8.630 to -1.925	Yes	***	0,0001
LFC vs. HFB	-5,508	-8.860 to -2.155	Yes	****	<0.0001
LFC vs. HFBN	-4,078	-7.430 to -0.7254	Yes	**	0,007
LFCN vs. LFB	-1,83	-5.093 to 1.433	No	ns	0,654
LFCN vs. LFBN	-0,7	-3.963 to 2.563	No	ns	0,9975
LFCN vs. HFC	-3,89	-7.153 to -0.6270	Yes	**	0,0089
LFCN vs. HFCN	-4,26	-7.523 to -0.9970	Yes	**	0,0028
LFCN vs. HFB	-4,49	-7.753 to -1.227	Yes	**	0,0013
LFCN vs. HFBN	-3,06	-6.323 to 0.2030	No	ns	0,0819
LFB vs. LFBN	1,13	-2.133 to 4.393	No	ns	0,9586
LFB vs. HFC	-2,06	-5.323 to 1.203	No	ns	0,5078
LFB vs. HFCN	-2,43	-5.693 to 0.8330	No	ns	0,2946
LFB vs. HFB	-2,66	-5.923 to 0.6030	No	ns	0,1937
LFB vs. HFBN	-1,23	-4.493 to 2.033	No	ns	0,9358
LFBN vs. HFC	-3,19	-6.453 to 0.07297	No	ns	0,06
LFBN vs. HFCN	-3,56	-6.823 to -0.2970	Yes	*	0,0228
LFBN vs. HFB	-3,79	-7.053 to -0.5270	Yes	*	0,0119
LFBN vs. HFBN	-2,36	-5.623 to 0.9030	No	ns	0,3307
HFC vs. HFCN	-0,37	-3.633 to 2.893	No	ns	>0.9999
HFC vs. HFB	-0,6	-3.863 to 2.663	No	ns	0,9991
HFC vs. HFBN	0,83	-2.433 to 4.093	No	ns	0,9929
HFCN vs. HFB	-0,23	-3.493 to 3.033	No	ns	>0.9999
HFCN vs. HFBN	1,2	-2.063 to 4.463	No	ns	0,9434
HFB vs. HFBN	1,43	-1.833 to 4.693	No	ns	0,8682

Week 7:

Tukey's multiple comparisons test	Mean Diff.	95.00% CI of diff.	Significant?	Summary	Adjusted P Value
LFC vs. LFCN	-0,48	-4.136 to 3.176	No	ns	>0.9999
LFC vs. LFB	-2,52	-6.176 to 1.136	No	ns	0,3923
LFC vs. LFBN	-1,21	-4.866 to 2.446	No	ns	0,9675
LFC vs. HFC	-5,44	-9.096 to -1.784	Yes	***	0,0004
LFC vs. HFCN	-5,43	-9.086 to -1.774	Yes	***	0,0004
LFC vs. HFB	-5,5	-9.251 to -1.749	Yes	***	0,0005
LFC vs. HFBN	-4,24	-7.896 to -0.5835	Yes	*	0,0121
LFCN vs. LFB	-2,04	-5.599 to 1.519	No	ns	0,6282
LFCN vs. LFBN	-0,73	-4.289 to 2.829	No	ns	0,9981
LFCN vs. HFC	-4,96	-8.519 to -1.401	Yes	**	0,0011
LFCN vs. HFCN	-4,95	-8.509 to -1.391	Yes	**	0,0011
LFCN vs. HFB	-5,02	-8.676 to -1.364	Yes	**	0,0014
LFCN vs. HFBN	-3,76	-7.319 to -0.2011	Yes	*	0,031
LFB vs. LFBN	1,31	-2.249 to 4.869	No	ns	0,943
LFB vs. HFC	-2,92	-6.479 to 0.6389	No	ns	0,1871
LFB vs. HFCN	-2,91	-6.469 to 0.6489	No	ns	0,1905
LFB vs. HFB	-2,98	-6.636 to 0.6765	No	ns	0,1938
LFB vs. HFBN	-1,72	-5.279 to 1.839	No	ns	0,7995
LFBN vs. HFC	-4,23	-7.789 to -0.6711	Yes	**	0,0092
LFBN vs. HFCN	-4,22	-7.779 to -0.6611	Yes	**	0,0095
LFBN vs. HFB	-4,29	-7.946 to -0.6335	Yes	*	0,0107
LFBN vs. HFBN	-3,03	-6.589 to 0.5289	No	ns	0,1526
HFC vs. HFCN	0,01	-3.549 to 3.569	No	ns	>0.9999
HFC vs. HFB	-0,06	-3.716 to 3.596	No	ns	>0.9999
HFC vs. HFBN	1,2	-2.359 to 4.759	No	ns	0,964
HFCN vs. HFB	-0,07	-3.726 to 3.586	No	ns	>0.9999
HFCN vs. HFBN	1,19	-2.369 to 4.749	No	ns	0,9656
HFB vs. HFBN	1,26	-2.396 to 4.916	No	ns	0,9596

Week 8:

Tukey's multiple comparisons test	Mean Diff.	95.00% CI of diff.	Significant?	Summary	Adjusted P Value
LFC vs. LFCN	-0,8978	-4.959 to 3.163	No	ns	0,997
LFC vs. LFB	-2,998	-7.059 to 1.063	No	ns	0,3049
LFC vs. LFBN	-1,498	-5.559 to 2.563	No	ns	0,9424
LFC vs. HFC	-6,628	-10.69 to -2.567	Yes	****	<0.0001
LFC vs. HFCN	-6,288	-10.35 to -2.227	Yes	***	0,0002
LFC vs. HFB	-6,533	-10.70 to -2.367	Yes	***	0,0002
LFC vs. HFBN	-5,028	-9.089 to -0.9669	Yes	**	0,0056
LFCN vs. LFB	-2,1	-6.053 to 1.853	No	ns	0,7123
LFCN vs. LFBN	-0,6	-4.553 to 3.353	No	ns	0,9997
LFCN vs. HFC	-5,73	-9.683 to -1.777	Yes	***	0,0006
LFCN vs. HFCN	-5,39	-9.343 to -1.437	Yes	**	0,0015
LFCN vs. HFB	-5,636	-9.696 to -1.575	Yes	**	0,0012
LFCN vs. HFBN	-4,13	-8.083 to -0.1774	Yes	*	0,0343
LFB vs. LFBN	1,5	-2.453 to 5.453	No	ns	0,9334
LFB vs. HFC	-3,63	-7.583 to 0.3226	No	ns	0,0948
LFB vs. HFCN	-3,29	-7.243 to 0.6626	No	ns	0,1732
LFB vs. HFB	-3,536	-7.596 to 0.5253	No	ns	0,1338
LFB vs. HFBN	-2,03	-5.983 to 1.923	No	ns	0,7459
LFBN vs. HFC	-5,13	-9.083 to -1.177	Yes	**	0,0031
LFBN vs. HFCN	-4,79	-8.743 to -0.8374	Yes	**	0,0073
LFBN vs. HFB	-5,036	-9.096 to -0.9747	Yes	**	0,0055
LFBN vs. HFBN	-3,53	-7.483 to 0.4226	No	ns	0,1141
HFC vs. HFCN	0,34	-3.613 to 4.293	No	ns	>0.9999
HFC vs. HFB	0,09444	-3.966 to 4.155	No	ns	>0.9999
HFC vs. HFBN	1,6	-2.353 to 5.553	No	ns	0,9086
HFCN vs. HFB	-0,2456	-4.306 to 3.815	No	ns	>0.9999
HFCN vs. HFBN	1,26	-2.693 to 5.213	No	ns	0,9736
HFB vs. HFBN	1,506	-2.555 to 5.566	No	ns	0,9408

Week 9:

Tukey's multiple comparisons test	Mean Diff.	95.00% CI of diff.	Significant?	Summary	Adjusted P Value
LFC vs. LFCN	-0,3444	-4.461 to 3.772	No	ns	>0.9999
LFC vs. LFB	-2,838	-6.850 to 1.174	No	ns	0,3583
LFC vs. LFBN	-1,608	-5.620 to 2.404	No	ns	0,9127
LFC vs. HFC	-7,088	-11.10 to -3.076	Yes	****	<0.0001
LFC vs. HFCN	-6,278	-10.29 to -2.266	Yes	***	0,0002
LFC vs. HFB	-6,567	-10.68 to -2.450	Yes	***	0,0001
LFC vs. HFBN	-5,098	-9.110 to -1.086	Yes	**	0,0041
LFCN vs. LFB	-2,493	-6.505 to 1.519	No	ns	0,5273
LFCN vs. LFBN	-1,263	-5.275 to 2.749	No	ns	0,9752
LFCN vs. HFC	-6,743	-10.76 to -2.731	Yes	****	<0.0001
LFCN vs. HFCN	-5,933	-9.945 to -1.921	Yes	***	0,0004
LFCN vs. HFB	-6,222	-10.34 to -2.106	Yes	***	0,0003
LFCN vs. HFBN	-4,753	-8.765 to - 0.7412	Yes	**	0,0096
LFB vs. LFBN	1,23	-2.675 to 5.135	No	ns	0,9752
LFB vs. HFC	-4,25	-8.155 to - 0.3449	Yes	*	0,0234
LFB vs. HFCN	-3,44	-7.345 to 0.4651	No	ns	0,1244
LFB vs. HFB	-3,729	-7.741 to 0.2833	No	ns	0,0872
LFB vs. HFBN	-2,26	-6.165 to 1.645	No	ns	0,6163
LFBN vs. HFC	-5,48	-9.385 to -1.575	Yes	**	0,001
LFBN vs. HFCN	-4,67	-8.575 to - 0.7649	Yes	**	0,0086
LFBN vs. HFB	-4,959	-8.971 to - 0.9467	Yes	**	0,0058
LFBN vs. HFBN	-3,49	-7.395 to 0.4151	No	ns	0,1135
HFC vs. HFCN	0,81	-3.095 to 4.715	No	ns	0,998
HFC vs. HFB	0,5211	-3.491 to 4.533	No	ns	>0.9999
HFC vs. HFBN	1,99	-1.915 to 5.895	No	ns	0,7529
HFCN vs. HFB	-0,2889	-4.301 to 3.723	No	ns	>0.9999
HFCN vs. HFBN	1,18	-2.725 to 5.085	No	ns	0,9804
HFB vs. HFBN	1,469	-2.543 to 5.481	No	ns	0,9444

Week 10:

Tukey's multiple comparisons test	Mean Diff.	95.00% CI of diff.	Significant?	Summary	Adjusted P Value
LFC vs. LFCN	-0,4667	-4.728 to 3.795	No	ns	>0.9999
LFC vs. LFB	-3,227	-7.380 to 0.9267	No	ns	0,2446
LFC vs. LFBN	-2,117	-6.270 to 2.037	No	ns	0,7528
LFC vs. HFC	-7,377	-11.53 to -3.223	Yes	****	<0.0001
LFC vs. HFCN	-6,637	-10.79 to -2.483	Yes	***	0,0001
LFC vs. HFB	-6,878	-11.14 to -2.616	Yes	****	<0.0001
LFC vs. HFBN	-5,807	-9.960 to -1.653	Yes	**	0,0011
LFCN vs. LFB	-2,76	-6.913 to 1.393	No	ns	0,4397
LFCN vs. LFBN	-1,65	-5.803 to 2.503	No	ns	0,9163
LFCN vs. HFC	-6,91	-11.06 to -2.757	Yes	****	<0.0001
LFCN vs. HFCN	-6,17	-10.32 to -2.017	Yes	***	0,0004
LFCN vs. HFB	-6,411	-10.67 to -2.150	Yes	***	0,0003
LFCN vs. HFBN	-5,34	-9.493 to -1.187	Yes	**	0,0035
LFB vs. LFBN	1,11	-2.933 to 5.153	No	ns	0,9887
LFB vs. HFC	-4,15	-8.193 to -0.1074	Yes	*	0,0401
LFB vs. HFCN	-3,41	-7.453 to 0.6326	No	ns	0,1608
LFB vs. HFB	-3,651	-7.805 to 0.5023	No	ns	0,126
LFB vs. HFBN	-2,58	-6.623 to 1.463	No	ns	0,4928
LFBN vs. HFC	-5,26	-9.303 to -1.217	Yes	**	0,003
LFBN vs. HFCN	-4,52	-8.563 to -0.4774	Yes	*	0,0179
LFBN vs. HFB	-4,761	-8.915 to -0.6077	Yes	*	0,0138
LFBN vs. HFBN	-3,69	-7.733 to 0.3526	No	ns	0,0988
HFC vs. HFCN	0,74	-3.303 to 4.783	No	ns	0,9991
HFC vs. HFB	0,4989	-3.655 to 4.652	No	ns	>0.9999
HFC vs. HFBN	1,57	-2.473 to 5.613	No	ns	0,9251
HFCN vs. HFB	-0,2411	-4.395 to 3.912	No	ns	>0.9999
HFCN vs. HFBN	0,83	-3.213 to 4.873	No	ns	0,9981
HFB vs. HFBN	1,071	-3.082 to 5.225	No	ns	0,9922

Week 11:

Tukey's multiple comparisons test	Mean Diff.	95.00% CI of diff.	Significant?	Summary	Adjusted P Value
LFC vs. LFCN	-0,5667	-5.093 to 3.960	No	ns	>0.9999
LFC vs. LFB	-3,511	-7.923 to 0.9010	No	ns	0,2182
LFC vs. LFBN	-2,271	-6.683 to 2.141	No	ns	0,7433
LFC vs. HFC	-7,651	-12.06 to -3.239	Yes	****	<0.0001
LFC vs. HFCN	-6,671	-11.08 to -2.259	Yes	***	0,0003
LFC vs. HFB	-7,633	-12.16 to -3.107	Yes	****	<0.0001
LFC vs. HFBN	-6,521	-10.93 to -2.109	Yes	***	0,0004
LFCN vs. LFB	-2,944	-7.357 to 1.468	No	ns	0,4341
LFCN vs. LFBN	-1,704	-6.117 to 2.708	No	ns	0,927
LFCN vs. HFC	-7,084	-11.50 to -2.672	Yes	***	0,0001
LFCN vs. HFCN	-6,104	-10.52 to -1.692	Yes	**	0,0012
LFCN vs. HFB	-7,067	-11.59 to -2.540	Yes	***	0,0002
LFCN vs. HFBN	-5,954	-10.37 to -1.542	Yes	**	0,0018
LFB vs. LFBN	1,24	-3.054 to 5.534	No	ns	0,9849
LFB vs. HFC	-4,14	-8.434 to 0.1545	No	ns	0,0668
LFB vs. HFCN	-3,16	-7.454 to 1.134	No	ns	0,3086
LFB vs. HFB	-4,122	-8.534 to 0.2899	No	ns	0,0841
LFB vs. HFBN	-3,01	-7.304 to 1.284	No	ns	0,37
LFBN vs. HFC	-5,38	-9.674 to -1.086	Yes	**	0,0049
LFBN vs. HFCN	-4,4	-8.694 to -0.1055	Yes	*	0,0408
LFBN vs. HFB	-5,362	-9.774 to -0.9501	Yes	**	0,0071
LFBN vs. HFBN	-4,25	-8.544 to 0.04445	No	ns	0,0544
HFC vs. HFCN	0,98	-3.314 to 5.274	No	ns	0,9963
HFC vs. HFB	0,01778	-4.394 to 4.430	No	ns	>0.9999
HFC vs. HFBN	1,13	-3.164 to 5.424	No	ns	0,9912
HFCN vs. HFB	-0,9622	-5.374 to 3.450	No	ns	0,9972
HFCN vs. HFBN	0,15	-4.144 to 4.444	No	ns	>0.9999
HFB vs. HFBN	1,112	-3.300 to 5.524	No	ns	0,9932

Week 12:

Tukey's multiple comparisons test	Mean Diff.	95.00% CI of diff.	Significant?	Summary	Adjusted P Value
LFC vs. LFCN	-0,6444	-5.623 to 4.334	No	ns	>0.9999
LFC vs. LFB	-3,989	-8.841 to 0.8635	No	ns	0,1851
LFC vs. LFBN	-2,359	-7.211 to 2.494	No	ns	0,7943
LFC vs. HFC	-8,029	-12.88 to -3.176	Yes	****	<0.0001
LFC vs. HFCN	-7,389	-12.24 to -2.536	Yes	***	0,0003
LFC vs. HFB	-7,944	-12.92 to -2.966	Yes	***	0,0001
LFC vs. HFBN	-6,459	-11.31 to -1.606	Yes	**	0,0022
LFCN vs. LFB	-3,344	-8.197 to 1.508	No	ns	0,3918
LFCN vs. LFBN	-1,714	-6.567 to 3.138	No	ns	0,9537
LFCN vs. HFC	-7,384	-12.24 to -2.532	Yes	***	0,0003
LFCN vs. HFCN	-6,744	-11.60 to -1.892	Yes	**	0,0012
LFCN vs. HFB	-7,3	-12.28 to -2.322	Yes	***	0,0005
LFCN vs. HFBN	-5,814	-10.67 to -0.9620	Yes	**	0,0084
LFB vs. LFBN	1,63	-3.093 to 6.353	No	ns	0,9591
LFB vs. HFC	-4,04	-8.763 to 0.6830	No	ns	0,1484
LFB vs. HFCN	-3,4	-8.123 to 1.323	No	ns	0,3359
LFB vs. HFB	-3,956	-8.808 to 0.8969	No	ns	0,1934
LFB vs. HFBN	-2,47	-7.193 to 2.253	No	ns	0,7278
LFBN vs. HFC	-5,67	-10.39 to -0.9470	Yes	**	0,0082
LFBN vs. HFCN	-5,03	-9.753 to -0.3070	Yes	*	0,0288
LFBN vs. HFB	-5,586	-10.44 to -0.7331	Yes	*	0,0132
LFBN vs. HFBN	-4,1	-8.823 to 0.6230	No	ns	0,136
HFC vs. HFCN	0,64	-4.083 to 5.363	No	ns	0,9999
HFC vs. HFB	0,08444	-4.768 to 4.937	No	ns	>0.9999
HFC vs. HFBN	1,57	-3.153 to 6.293	No	ns	0,9666
HFCN vs. HFB	-0,5556	-5.408 to 4.297	No	ns	>0.9999
HFCN vs. HFBN	0,93	-3.793 to 5.653	No	ns	0,9986
HFB vs. HFBN	1,486	-3.367 to 6.338	No	ns	0,9788
