# Supplementary material for: Dietary pH Enhancement Improves Metabolic Outcomes in Diet-Induced Obese Male and Female Mice: Effects of Beef vs. Casein Proteins
Source: Nutrients. 2022 Jun 22;14(13):2583. doi: 10.3390/nu14132583 (PMC9268221; doi:10.3390/nu14132583)
Supplement: Supplementary file 1 [file nutrients-14-02583-s001.zip › Supplementary Table S2B_Female Weekly Body Weights.pdf]

Supplementary Table S2B: The effects of dietary fat, protein source and pH enhancement in weekly body weight in female mice.

Legends: LFC, low fat casein; LFCN, low fat casein pH enhanced; LFB, low fat beef; LFBN, low fat beef pH enhanced; HFC, high fat casein; HFCN, high fat casein pH enhanced; HFB, high fat beef; HFBN, high fat beef pH enhanced.

Week 0:

Tukey's multiple comparisons test	Mean Diff.	95.00% CI of diff.	Significant?	Summary	Adjusted P Value
LFC vs. LFCN	0,02	-3.108 to 3.148	No	ns	>0.9999
LFC vs. LFB	-0,56	-3.269 to 2.149	No	ns	0,9976
LFC vs. LFBN	-0,71	-3.419 to 1.999	No	ns	0,9898
LFC vs. HFC	0,82	-2.308 to 3.948	No	ns	0,9898
LFC vs. HFCN	-0,5	-3.628 to 2.628	No	ns	0,9995
LFC vs. HFB	0,97	-2.348 to 4.288	No	ns	0,9808
LFC vs. HFBN	0,64	-2.488 to 3.768	No	ns	0,9978
LFCN vs. LFB	-0,58	-3.289 to 2.129	No	ns	0,997
LFCN vs. LFBN	-0,73	-3.439 to 1.979	No	ns	0,988
LFCN vs. HFC	0,8	-2.328 to 3.928	No	ns	0,9912
LFCN vs. HFCN	-0,52	-3.648 to 2.608	No	ns	0,9994
LFCN vs. HFB	0,95	-2.368 to 4.268	No	ns	0,983
LFCN vs. HFBN	0,62	-2.508 to 3.748	No	ns	0,9982
LFB vs. LFBN	-0,15	-2.362 to 2.062	No	ns	>0.9999
LFB vs. HFC	1,38	-1.329 to 4.089	No	ns	0,7322
LFB vs. HFCN	0,06	-2.649 to 2.769	No	ns	>0.9999
LFB vs. HFB	1,53	-1.396 to 4.456	No	ns	0,7062
LFB vs. HFBN	1,2	-1.509 to 3.909	No	ns	0,8456
LFBN vs. HFC	1,53	-1.179 to 4.239	No	ns	0,6218
LFBN vs. HFCN	0,21	-2.499 to 2.919	No	ns	>0.9999
LFBN vs. HFB	1,68	-1.246 to 4.606	No	ns	0,6024
LFBN vs. HFBN	1,35	-1.359 to 4.059	No	ns	0,7529
HFC vs. HFCN	-1,32	-4.448 to 1.808	No	ns	0,875
HFC vs. HFB	0,15	-3.168 to 3.468	No	ns	>0.9999
HFC vs. HFBN	-0,18	-3.308 to 2.948	No	ns	>0.9999
HFCN vs. HFB	1,47	-1.848 to 4.788	No	ns	0,8454
HFCN vs. HFBN	1,14	-1.988 to 4.268	No	ns	0,9377
HFB vs. HFBN	-0,33	-3.648 to 2.988	No	ns	>0.9999

Week 1:

Tukey's multiple comparisons test	Mean Diff.	95.00% CI of diff.	Significant?	Summary	Adjusted P Value
LFC vs. LFCN	-0,4	-2.333 to 1.533	No	ns	0,998
LFC vs. LFB	-0,76	-2.693 to 1.173	No	ns	0,9211
LFC vs. LFBN	-0,27	-2.203 to 1.663	No	ns	0,9999
LFC vs. HFC	0,02	-1.913 to 1.953	No	ns	>0.9999
LFC vs. HFCN	-0,02	-1.953 to 1.913	No	ns	>0.9999
LFC vs. HFB	0,24	-1.693 to 2.173	No	ns	>0.9999
LFC vs. HFBN	-0,25	-2.183 to 1.683	No	ns	>0.9999
LFCN vs. LFB	-0,36	-2.293 to 1.573	No	ns	0,999
LFCN vs. LFBN	0,13	-1.803 to 2.063	No	ns	>0.9999
LFCN vs. HFC	0,42	-1.513 to 2.353	No	ns	0,9973
LFCN vs. HFCN	0,38	-1.553 to 2.313	No	ns	0,9986
LFCN vs. HFB	0,64	-1.293 to 2.573	No	ns	0,9677
LFCN vs. HFBN	0,15	-1.783 to 2.083	No	ns	>0.9999
LFB vs. LFBN	0,49	-1.443 to 2.423	No	ns	0,9931
LFB vs. HFC	0,78	-1.153 to 2.713	No	ns	0,9105
LFB vs. HFCN	0,74	-1.193 to 2.673	No	ns	0,9309
LFB vs. HFB	1	-0.9335 to 2.933	No	ns	0,74
LFB vs. HFBN	0,51	-1.423 to 2.443	No	ns	0,9912
LFBN vs. HFC	0,29	-1.643 to 2.223	No	ns	0,9998
LFBN vs. HFCN	0,25	-1.683 to 2.183	No	ns	>0.9999
LFBN vs. HFB	0,51	-1.423 to 2.443	No	ns	0,9912
LFBN vs. HFBN	0,02	-1.913 to 1.953	No	ns	>0.9999
HFC vs. HFCN	-0,04	-1.973 to 1.893	No	ns	>0.9999
HFC vs. HFB	0,22	-1.713 to 2.153	No	ns	>0.9999
HFC vs. HFBN	-0,27	-2.203 to 1.663	No	ns	0,9999
HFCN vs. HFB	0,26	-1.673 to 2.193	No	ns	0,9999
HFCN vs. HFBN	-0,23	-2.163 to 1.703	No	ns	>0.9999
HFB vs. HFBN	-0,49	-2.423 to 1.443	No	ns	0,9931

Week 2:

Tukey's multiple comparisons test	Mean Diff.	95.00% CI of diff.	Significant?	Summary	Adjusted P Value
LFC vs. LFCN	-0,05	-1.711 to 1.611	No	ns	>0.9999
LFC vs. LFB	-0,61	-2.271 to 1.051	No	ns	0,9439
LFC vs. LFBN	-0,56	-2.221 to 1.101	No	ns	0,9643
LFC vs. HFC	-0,19	-1.851 to 1.471	No	ns	>0.9999
LFC vs. HFCN	-0,04	-1.701 to 1.621	No	ns	>0.9999
LFC vs. HFB	-0,09	-1.751 to 1.571	No	ns	>0.9999
LFC vs. HFBN	-0,51	-2.171 to 1.151	No	ns	0,9787
LFCN vs. LFB	-0,56	-2.221 to 1.101	No	ns	0,9643
LFCN vs. LFBN	-0,51	-2.171 to 1.151	No	ns	0,9787
LFCN vs. HFC	-0,14	-1.801 to 1.521	No	ns	>0.9999
LFCN vs. HFCN	0,01	-1.651 to 1.671	No	ns	>0.9999
LFCN vs. HFB	-0,04	-1.701 to 1.621	No	ns	>0.9999
LFCN vs. HFBN	-0,46	-2.121 to 1.201	No	ns	0,9883
LFB vs. LFBN	0,05	-1.611 to 1.711	No	ns	>0.9999
LFB vs. HFC	0,42	-1.241 to 2.081	No	ns	0,9932
LFB vs. HFCN	0,57	-1.091 to 2.231	No	ns	0,9607
LFB vs. HFB	0,52	-1.141 to 2.181	No	ns	0,9762
LFB vs. HFBN	0,1	-1.561 to 1.761	No	ns	>0.9999
LFBN vs. HFC	0,37	-1.291 to 2.031	No	ns	0,9969
LFBN vs. HFCN	0,52	-1.141 to 2.181	No	ns	0,9762
LFBN vs. HFB	0,47	-1.191 to 2.131	No	ns	0,9867
LFBN vs. HFBN	0,05	-1.611 to 1.711	No	ns	>0.9999
HFC vs. HFCN	0,15	-1.511 to 1.811	No	ns	>0.9999
HFC vs. HFB	0,1	-1.561 to 1.761	No	ns	>0.9999
HFC vs. HFBN	-0,32	-1.981 to 1.341	No	ns	0,9988
HFCN vs. HFB	-0,05	-1.711 to 1.611	No	ns	>0.9999
HFCN vs. HFBN	-0,47	-2.131 to 1.191	No	ns	0,9867
HFB vs. HFBN	-0,42	-2.081 to 1.241	No	ns	0,9932

Week 3:

Tukey's multiple comparisons test	Mean Diff.	95.00% CI of diff.	Significant?	Summary	Adjusted P Value
LFC vs. LFCN	-0,12	-1.646 to 1.406	No	ns	>0.9999
LFC vs. LFB	-0,9	-2.426 to 0.6255	No	ns	0,5944
LFC vs. LFBN	-0,48	-2.006 to 1.046	No	ns	0,9756
LFC vs. HFC	-0,51	-2.036 to 1.016	No	ns	0,9659
LFC vs. HFCN	-0,02	-1.546 to 1.506	No	ns	>0.9999
LFC vs. HFB	0,17	-1.356 to 1.696	No	ns	>0.9999
LFC vs. HFBN	-0,15	-1.676 to 1.376	No	ns	>0.9999
LFCN vs. LFB	-0,78	-2.306 to 0.7455	No	ns	0,751
LFCN vs. LFBN	-0,36	-1.886 to 1.166	No	ns	0,9955
LFCN vs. HFC	-0,39	-1.916 to 1.136	No	ns	0,9927
LFCN vs. HFCN	0,1	-1.426 to 1.626	No	ns	>0.9999
LFCN vs. HFB	0,29	-1.236 to 1.816	No	ns	0,9989
LFCN vs. HFBN	-0,03	-1.556 to 1.496	No	ns	>0.9999
LFB vs. LFBN	0,42	-1.106 to 1.946	No	ns	0,9887
LFB vs. HFC	0,39	-1.136 to 1.916	No	ns	0,9927
LFB vs. HFCN	0,88	-0.6455 to 2.406	No	ns	0,6216
LFB vs. HFB	1,07	-0.4555 to 2.596	No	ns	0,3702
LFB vs. HFBN	0,75	-0.7755 to 2.276	No	ns	0,7861
LFBN vs. HFC	-0,03	-1.556 to 1.496	No	ns	>0.9999
LFBN vs. HFCN	0,46	-1.066 to 1.986	No	ns	0,9808
LFBN vs. HFB	0,65	-0.8755 to 2.176	No	ns	0,8843
LFBN vs. HFBN	0,33	-1.196 to 1.856	No	ns	0,9974
HFC vs. HFCN	0,49	-1.036 to 2.016	No	ns	0,9726
HFC vs. HFB	0,68	-0.8455 to 2.206	No	ns	0,8582
HFC vs. HFBN	0,36	-1.166 to 1.886	No	ns	0,9955
HFCN vs. HFB	0,19	-1.336 to 1.716	No	ns	>0.9999
HFCN vs. HFBN	-0,13	-1.656 to 1.396	No	ns	>0.9999
HFB vs. HFBN	-0,32	-1.846 to 1.206	No	ns	0,9979

Week 4:

Tukey's multiple comparisons test	Mean Diff.	95.00% CI of diff.	Significant?	Summary	Adjusted P Value
LFC vs. LFCN	-0,41	-2.108 to 1.288	No	ns	0,9949
LFC vs. LFB	-1,33	-3.028 to 0.3678	No	ns	0,236
LFC vs. LFBN	-0,96	-2.658 to 0.7378	No	ns	0,6451
LFC vs. HFC	-1,1	-2.798 to 0.5978	No	ns	0,4744
LFC vs. HFCN	-0,55	-2.248 to 1.148	No	ns	0,9713
LFC vs. HFB	-0,48	-2.178 to 1.218	No	ns	0,9868
LFC vs. HFBN	-1,03	-2.728 to 0.6678	No	ns	0,5595
LFCN vs. LFB	-0,92	-2.618 to 0.7778	No	ns	0,6925
LFCN vs. LFBN	-0,55	-2.248 to 1.148	No	ns	0,9713
LFCN vs. HFC	-0,69	-2.388 to 1.008	No	ns	0,9073
LFCN vs. HFCN	-0,14	-1.838 to 1.558	No	ns	>0.9999
LFCN vs. HFB	-0,07	-1.768 to 1.628	No	ns	>0.9999
LFCN vs. HFBN	-0,62	-2.318 to 1.078	No	ns	0,9455
LFB vs. LFBN	0,37	-1.328 to 2.068	No	ns	0,9973
LFB vs. HFC	0,23	-1.468 to 1.928	No	ns	0,9999
LFB vs. HFCN	0,78	-0.9178 to 2.478	No	ns	0,8383
LFB vs. HFB	0,85	-0.8478 to 2.548	No	ns	0,7703
LFB vs. HFBN	0,3	-1.398 to 1.998	No	ns	0,9993
LFBN vs. HFC	-0,14	-1.838 to 1.558	No	ns	>0.9999
LFBN vs. HFCN	0,41	-1.288 to 2.108	No	ns	0,9949
LFBN vs. HFB	0,48	-1.218 to 2.178	No	ns	0,9868
LFBN vs. HFBN	-0,07	-1.768 to 1.628	No	ns	>0.9999
HFC vs. HFCN	0,55	-1.148 to 2.248	No	ns	0,9713
HFC vs. HFB	0,62	-1.078 to 2.318	No	ns	0,9455
HFC vs. HFBN	0,07	-1.628 to 1.768	No	ns	>0.9999
HFCN vs. HFB	0,07	-1.628 to 1.768	No	ns	>0.9999
HFCN vs. HFBN	-0,48	-2.178 to 1.218	No	ns	0,9868
HFB vs. HFBN	-0,55	-2.248 to 1.148	No	ns	0,9713

Week 5:

Tukey's multiple comparisons test	Mean Diff.	95.00% CI of diff.	Significant?	Summary	Adjusted P Value
LFC vs. LFCN	-0,46	-2.348 to 1.428	No	ns	0,9946
LFC vs. LFB	-1,4	-3.288 to 0.4878	No	ns	0,3
LFC vs. LFBN	-0,95	-2.838 to 0.9378	No	ns	0,7657
LFC vs. HFC	-0,78	-2.668 to 1.108	No	ns	0,8997
LFC vs. HFCN	-0,51	-2.398 to 1.378	No	ns	0,9899
LFC vs. HFB	-0,56	-2.448 to 1.328	No	ns	0,9825
LFC vs. HFBN	-0,63	-2.518 to 1.258	No	ns	0,9662
LFCN vs. LFB	-0,94	-2.828 to 0.9478	No	ns	0,7751
LFCN vs. LFBN	-0,49	-2.378 to 1.398	No	ns	0,992
LFCN vs. HFC	-0,32	-2.208 to 1.568	No	ns	0,9995
LFCN vs. HFCN	-0,05	-1.938 to 1.838	No	ns	>0.9999
LFCN vs. HFB	-0,1	-1.988 to 1.788	No	ns	>0.9999
LFCN vs. HFBN	-0,17	-2.058 to 1.718	No	ns	>0.9999
LFB vs. LFBN	0,45	-1.438 to 2.338	No	ns	0,9952
LFB vs. HFC	0,62	-1.268 to 2.508	No	ns	0,969
LFB vs. HFCN	0,89	-0.9978 to 2.778	No	ns	0,8196
LFB vs. HFB	0,84	-1.048 to 2.728	No	ns	0,8593
LFB vs. HFBN	0,77	-1.118 to 2.658	No	ns	0,9057
LFBN vs. HFC	0,17	-1.718 to 2.058	No	ns	>0.9999
LFBN vs. HFCN	0,44	-1.448 to 2.328	No	ns	0,9959
LFBN vs. HFB	0,39	-1.498 to 2.278	No	ns	0,9981
LFBN vs. HFBN	0,32	-1.568 to 2.208	No	ns	0,9995
HFC vs. HFCN	0,27	-1.618 to 2.158	No	ns	0,9998
HFC vs. HFB	0,22	-1.668 to 2.108	No	ns	>0.9999
HFC vs. HFBN	0,15	-1.738 to 2.038	No	ns	>0.9999
HFCN vs. HFB	-0,05	-1.938 to 1.838	No	ns	>0.9999
HFCN vs. HFBN	-0,12	-2.008 to 1.768	No	ns	>0.9999
HFB vs. HFBN	-0,07	-1.958 to 1.818	No	ns	>0.9999

Week 6:

Tukey's multiple comparisons test	Mean Diff.	95.00% CI of diff.	Significant?	Summary	Adjusted P Value
LFC vs. LFCN	-0,19	-2.149 to 1.769	No	ns	>0.9999
LFC vs. LFB	-1,2	-3.159 to 0.7590	No	ns	0,5473
LFC vs. LFBN	-0,66	-2.619 to 1.299	No	ns	0,9644
LFC vs. HFC	-0,87	-2.829 to 1.089	No	ns	0,8605
LFC vs. HFCN	-0,42	-2.379 to 1.539	No	ns	0,9975
LFC vs. HFB	-0,38	-2.339 to 1.579	No	ns	0,9987
LFC vs. HFBN	-1,39	-3.349 to 0.5690	No	ns	0,3554
LFCN vs. LFB	-1,01	-2.969 to 0.9490	No	ns	0,7431
LFCN vs. LFBN	-0,47	-2.429 to 1.489	No	ns	0,9951
LFCN vs. HFC	-0,68	-2.639 to 1.279	No	ns	0,9583
LFCN vs. HFCN	-0,23	-2.189 to 1.729	No	ns	>0.9999
LFCN vs. HFB	-0,19	-2.149 to 1.769	No	ns	>0.9999
LFCN vs. HFBN	-1,2	-3.159 to 0.7590	No	ns	0,5473
LFB vs. LFBN	0,54	-1.419 to 2.499	No	ns	0,9886
LFB vs. HFC	0,33	-1.629 to 2.289	No	ns	0,9995
LFB vs. HFCN	0,78	-1.179 to 2.739	No	ns	0,916
LFB vs. HFB	0,82	-1.139 to 2.779	No	ns	0,8934
LFB vs. HFBN	-0,19	-2.149 to 1.769	No	ns	>0.9999
LFBN vs. HFC	-0,21	-2.169 to 1.749	No	ns	>0.9999
LFBN vs. HFCN	0,24	-1.719 to 2.199	No	ns	>0.9999
LFBN vs. HFB	0,28	-1.679 to 2.239	No	ns	0,9998
LFBN vs. HFBN	-0,73	-2.689 to 1.229	No	ns	0,9396
HFC vs. HFCN	0,45	-1.509 to 2.409	No	ns	0,9962
HFC vs. HFB	0,49	-1.469 to 2.449	No	ns	0,9936
HFC vs. HFBN	-0,52	-2.479 to 1.439	No	ns	0,9909
HFCN vs. HFB	0,04	-1.919 to 1.999	No	ns	>0.9999
HFCN vs. HFBN	-0,97	-2.929 to 0.9890	No	ns	0,78
HFB vs. HFBN	-1,01	-2.969 to 0.9490	No	ns	0,7431

Week 7:

Tukey's multiple comparisons test	Mean Diff.	95.00% CI of diff.	Significant?	Summary	Adjusted P Value
LFC vs. LFCN	-0,11	-2.318 to 2.098	No	ns	>0.9999
LFC vs. LFB	-1,01	-3.218 to 1.198	No	ns	0,8412
LFC vs. LFBN	-0,82	-3.028 to 1.388	No	ns	0,9406
LFC vs. HFC	-0,85	-3.058 to 1.358	No	ns	0,9288
LFC vs. HFCN	-0,27	-2.478 to 1.938	No	ns	>0.9999
LFC vs. HFB	-0,06	-2.268 to 2.148	No	ns	>0.9999
LFC vs. HFBN	-0,62	-2.828 to 1.588	No	ns	0,9873
LFCN vs. LFB	-0,9	-3.108 to 1.308	No	ns	0,9059
LFCN vs. LFBN	-0,71	-2.918 to 1.498	No	ns	0,9724
LFCN vs. HFC	-0,74	-2.948 to 1.468	No	ns	0,9654
LFCN vs. HFCN	-0,16	-2.368 to 2.048	No	ns	>0.9999
LFCN vs. HFB	0,05	-2.158 to 2.258	No	ns	>0.9999
LFCN vs. HFBN	-0,51	-2.718 to 1.698	No	ns	0,9961
LFB vs. LFBN	0,19	-2.018 to 2.398	No	ns	>0.9999
LFB vs. HFC	0,16	-2.048 to 2.368	No	ns	>0.9999
LFB vs. HFCN	0,74	-1.468 to 2.948	No	ns	0,9654
LFB vs. HFB	0,95	-1.258 to 3.158	No	ns	0,8789
LFB vs. HFBN	0,39	-1.818 to 2.598	No	ns	0,9993
LFBN vs. HFC	-0,03	-2.238 to 2.178	No	ns	>0.9999
LFBN vs. HFCN	0,55	-1.658 to 2.758	No	ns	0,9938
LFBN vs. HFB	0,76	-1.448 to 2.968	No	ns	0,9601
LFBN vs. HFBN	0,2	-2.008 to 2.408	No	ns	>0.9999
HFC vs. HFCN	0,58	-1.628 to 2.788	No	ns	0,9914
HFC vs. HFB	0,79	-1.418 to 2.998	No	ns	0,951
HFC vs. HFBN	0,23	-1.978 to 2.438	No	ns	>0.9999
HFCN vs. HFB	0,21	-1.998 to 2.418	No	ns	>0.9999
HFCN vs. HFBN	-0,35	-2.558 to 1.858	No	ns	0,9997
HFB vs. HFBN	-0,56	-2.768 to 1.648	No	ns	0,9931

Week 8:

Tukey's multiple comparisons test	Mean Diff.	95.00% CI of diff.	Significant?	Summary	Adjusted P Value
LFC vs. LFCN	-0,1	-2.138 to 1.938	No	ns	>0.9999
LFC vs. LFB	-0,66	-2.698 to 1.378	No	ns	0,9713
LFC vs. LFBN	-0,25	-2.288 to 1.788	No	ns	>0.9999
LFC vs. HFC	-1,19	-3.228 to 0.8476	No	ns	0,6067
LFC vs. HFCN	-0,24	-2.278 to 1.798	No	ns	>0.9999
LFC vs. HFB	-0,34	-2.378 to 1.698	No	ns	0,9995
LFC vs. HFBN	-0,34	-2.378 to 1.698	No	ns	0,9995
LFCN vs. LFB	-0,56	-2.598 to 1.478	No	ns	0,9888
LFCN vs. LFBN	-0,15	-2.188 to 1.888	No	ns	>0.9999
LFCN vs. HFC	-1,09	-3.128 to 0.9476	No	ns	0,7061
LFCN vs. HFCN	-0,14	-2.178 to 1.898	No	ns	>0.9999
LFCN vs. HFB	-0,24	-2.278 to 1.798	No	ns	>0.9999
LFCN vs. HFBN	-0,24	-2.278 to 1.798	No	ns	>0.9999
LFB vs. LFBN	0,41	-1.628 to 2.448	No	ns	0,9984
LFB vs. HFC	-0,53	-2.568 to 1.508	No	ns	0,9919
LFB vs. HFCN	0,42	-1.618 to 2.458	No	ns	0,9981
LFB vs. HFB	0,32	-1.718 to 2.358	No	ns	0,9997
LFB vs. HFBN	0,32	-1.718 to 2.358	No	ns	0,9997
LFBN vs. HFC	-0,94	-2.978 to 1.098	No	ns	0,8354
LFBN vs. HFCN	0,01	-2.028 to 2.048	No	ns	>0.9999
LFBN vs. HFB	-0,09	-2.128 to 1.948	No	ns	>0.9999
LFBN vs. HFBN	-0,09	-2.128 to 1.948	No	ns	>0.9999
HFC vs. HFCN	0,95	-1.088 to 2.988	No	ns	0,8278
HFC vs. HFB	0,85	-1.188 to 2.888	No	ns	0,8951
HFC vs. HFBN	0,85	-1.188 to 2.888	No	ns	0,8951
HFCN vs. HFB	-0,1	-2.138 to 1.938	No	ns	>0.9999
HFCN vs. HFBN	-0,1	-2.138 to 1.938	No	ns	>0.9999
HFB vs. HFBN	0	-2.038 to 2.038	No	ns	>0.9999

Week 9:

Tukey's multiple comparisons test	Mean Diff.	95.00% CI of diff.	Significant?	Summary	Adjusted P Value
LFC vs. LFCN	0,22	-2.287 to 2.727	No	ns	>0.9999
LFC vs. LFB	-1,28	-3.787 to 1.227	No	ns	0,7523
LFC vs. LFBN	-0,6	-3.107 to 1.907	No	ns	0,9951
LFC vs. HFC	-1,2	-3.707 to 1.307	No	ns	0,8079
LFC vs. HFCN	-0,69	-3.197 to 1.817	No	ns	0,9887
LFC vs. HFB	-0,62	-3.127 to 1.887	No	ns	0,994
LFC vs. HFBN	-0,6	-3.107 to 1.907	No	ns	0,9951
LFCN vs. LFB	-1,5	-4.007 to 1.007	No	ns	0,5769
LFCN vs. LFBN	-0,82	-3.327 to 1.687	No	ns	0,9697
LFCN vs. HFC	-1,42	-3.927 to 1.087	No	ns	0,643
LFCN vs. HFCN	-0,91	-3.417 to 1.597	No	ns	0,9472
LFCN vs. HFB	-0,84	-3.347 to 1.667	No	ns	0,9655
LFCN vs. HFBN	-0,82	-3.327 to 1.687	No	ns	0,9697
LFB vs. LFBN	0,68	-1.827 to 3.187	No	ns	0,9896
LFB vs. HFC	0,08	-2.427 to 2.587	No	ns	>0.9999
LFB vs. HFCN	0,59	-1.917 to 3.097	No	ns	0,9956
LFB vs. HFB	0,66	-1.847 to 3.167	No	ns	0,9913
LFB vs. HFBN	0,68	-1.827 to 3.187	No	ns	0,9896
LFBN vs. HFC	-0,6	-3.107 to 1.907	No	ns	0,9951
LFBN vs. HFCN	-0,09	-2.597 to 2.417	No	ns	>0.9999
LFBN vs. HFB	-0,02	-2.527 to 2.487	No	ns	>0.9999
LFBN vs. HFBN	0	-2.507 to 2.507	No	ns	>0.9999
HFC vs. HFCN	0,51	-1.997 to 3.017	No	ns	0,9982
HFC vs. HFB	0,58	-1.927 to 3.087	No	ns	0,996
HFC vs. HFBN	0,6	-1.907 to 3.107	No	ns	0,9951
HFCN vs. HFB	0,07	-2.437 to 2.577	No	ns	>0.9999
HFCN vs. HFBN	0,09	-2.417 to 2.597	No	ns	>0.9999
HFB vs. HFBN	0,02	-2.487 to 2.527	No	ns	>0.9999

Week 10:

Tukey's multiple comparisons test	Mean Diff.	95.00% CI of diff.	Significant?	Summary	Adjusted P Value
LFC vs. LFCN	0,12	-2.525 to 2.765	No	ns	>0.9999
LFC vs. LFB	-1,66	-4.305 to 0.9846	No	ns	0,5158
LFC vs. LFBN	-1,15	-3.795 to 1.495	No	ns	0,873
LFC vs. HFC	-1,42	-4.065 to 1.225	No	ns	0,7022
LFC vs. HFCN	-0,99	-3.635 to 1.655	No	ns	0,9382
LFC vs. HFB	-0,52	-3.165 to 2.125	No	ns	0,9986
LFC vs. HFBN	-0,52	-3.165 to 2.125	No	ns	0,9986
LFCN vs. LFB	-1,78	-4.425 to 0.8646	No	ns	0,4241
LFCN vs. LFBN	-1,27	-3.915 to 1.375	No	ns	0,8054
LFCN vs. HFC	-1,54	-4.185 to 1.105	No	ns	0,6102
LFCN vs. HFCN	-1,11	-3.755 to 1.535	No	ns	0,892
LFCN vs. HFB	-0,64	-3.285 to 2.005	No	ns	0,9948
LFCN vs. HFBN	-0,64	-3.285 to 2.005	No	ns	0,9948
LFB vs. LFBN	0,51	-2.135 to 3.155	No	ns	0,9988
LFB vs. HFC	0,24	-2.405 to 2.885	No	ns	>0.9999
LFB vs. HFCN	0,67	-1.975 to 3.315	No	ns	0,9931
LFB vs. HFB	1,14	-1.505 to 3.785	No	ns	0,8779
LFB vs. HFBN	1,14	-1.505 to 3.785	No	ns	0,8779
LFBN vs. HFC	-0,27	-2.915 to 2.375	No	ns	>0.9999
LFBN vs. HFCN	0,16	-2.485 to 2.805	No	ns	>0.9999
LFBN vs. HFB	0,63	-2.015 to 3.275	No	ns	0,9953
LFBN vs. HFBN	0,63	-2.015 to 3.275	No	ns	0,9953
HFC vs. HFCN	0,43	-2.215 to 3.075	No	ns	0,9996
HFC vs. HFB	0,9	-1.745 to 3.545	No	ns	0,9624
HFC vs. HFBN	0,9	-1.745 to 3.545	No	ns	0,9624
HFCN vs. HFB	0,47	-2.175 to 3.115	No	ns	0,9993
HFCN vs. HFBN	0,47	-2.175 to 3.115	No	ns	0,9993
HFB vs. HFBN	0	-2.645 to 2.645	No	ns	>0.9999

Week 11:

Tukey's multiple comparisons test	Mean Diff.	95.00% CI of diff.	Significant?	Summary	Adjusted P Value
LFC vs. LFCN	0,19	-2.616 to 2.996	No	ns	>0.9999
LFC vs. LFB	-1,32	-4.126 to 1.486	No	ns	0,8212
LFC vs. LFBN	-0,94	-3.746 to 1.866	No	ns	0,9655
LFC vs. HFC	-2,06	-4.866 to 0.7456	No	ns	0,3122
LFC vs. HFCN	-1,19	-3.996 to 1.616	No	ns	0,8867
LFC vs. HFB	-1,08	-3.886 to 1.726	No	ns	0,9289
LFC vs. HFBN	-0,78	-3.586 to 2.026	No	ns	0,988
LFCN vs. LFB	-1,51	-4.316 to 1.296	No	ns	0,6997
LFCN vs. LFBN	-1,13	-3.936 to 1.676	No	ns	0,9112
LFCN vs. HFC	-2,25	-5.056 to 0.5556	No	ns	0,2107
LFCN vs. HFCN	-1,38	-4.186 to 1.426	No	ns	0,7857
LFCN vs. HFB	-1,27	-4.076 to 1.536	No	ns	0,8483
LFCN vs. HFBN	-0,97	-3.776 to 1.836	No	ns	0,9591
LFB vs. LFBN	0,38	-2.426 to 3.186	No	ns	0,9999
LFB vs. HFC	-0,74	-3.546 to 2.066	No	ns	0,9912
LFB vs. HFCN	0,13	-2.676 to 2.936	No	ns	>0.9999
LFB vs. HFB	0,24	-2.566 to 3.046	No	ns	>0.9999
LFB vs. HFBN	0,54	-2.266 to 3.346	No	ns	0,9988
LFBN vs. HFC	-1,12	-3.926 to 1.686	No	ns	0,9149
LFBN vs. HFCN	-0,25	-3.056 to 2.556	No	ns	>0.9999
LFBN vs. HFB	-0,14	-2.946 to 2.666	No	ns	>0.9999
LFBN vs. HFBN	0,16	-2.646 to 2.966	No	ns	>0.9999
HFC vs. HFCN	0,87	-1.936 to 3.676	No	ns	0,9775
HFC vs. HFB	0,98	-1.826 to 3.786	No	ns	0,9568
HFC vs. HFBN	1,28	-1.526 to 4.086	No	ns	0,843
HFCN vs. HFB	0,11	-2.696 to 2.916	No	ns	>0.9999
HFCN vs. HFBN	0,41	-2.396 to 3.216	No	ns	0,9998
HFB vs. HFBN	0,3	-2.506 to 3.106	No	ns	>0.9999

Week 12:

Tukey's multiple comparisons test	Mean Diff.	95.00% CI of diff.	Significant?	Summary	Adjusted P Value
LFC vs. LFCN	-0,27	-3.473 to 2.933	No	ns	>0.9999
LFC vs. LFB	-2,52	-5.723 to 0.6833	No	ns	0,2314
LFC vs. LFBN	-1,92	-5.123 to 1.283	No	ns	0,5748
LFC vs. HFC	-2,99	-6.193 to 0.2133	No	ns	0,0848
LFC vs. HFCN	-1,92	-5.123 to 1.283	No	ns	0,5748
LFC vs. HFB	-1,6	-4.803 to 1.603	No	ns	0,7724
LFC vs. HFBN	-1,83	-5.033 to 1.373	No	ns	0,6331
LFCN vs. LFB	-2,25	-5.453 to 0.9533	No	ns	0,3684
LFCN vs. LFBN	-1,65	-4.853 to 1.553	No	ns	0,744
LFCN vs. HFC	-2,72	-5.923 to 0.4833	No	ns	0,1553
LFCN vs. HFCN	-1,65	-4.853 to 1.553	No	ns	0,744
LFCN vs. HFB	-1,33	-4.533 to 1.873	No	ns	0,8974
LFCN vs. HFBN	-1,56	-4.763 to 1.643	No	ns	0,794
LFB vs. LFBN	0,6	-2.603 to 3.803	No	ns	0,999
LFB vs. HFC	-0,47	-3.673 to 2.733	No	ns	0,9998
LFB vs. HFCN	0,6	-2.603 to 3.803	No	ns	0,999
LFB vs. HFB	0,92	-2.283 to 4.123	No	ns	0,9855
LFB vs. HFBN	0,69	-2.513 to 3.893	No	ns	0,9975
LFBN vs. HFC	-1,07	-4.273 to 2.133	No	ns	0,966
LFBN vs. HFCN	0	-3.203 to 3.203	No	ns	>0.9999
LFBN vs. HFB	0,32	-2.883 to 3.523	No	ns	>0.9999
LFBN vs. HFBN	0,09	-3.113 to 3.293	No	ns	>0.9999
HFC vs. HFCN	1,07	-2.133 to 4.273	No	ns	0,966
HFC vs. HFB	1,39	-1.813 to 4.593	No	ns	0,8742
HFC vs. HFBN	1,16	-2.043 to 4.363	No	ns	0,9479
HFCN vs. HFB	0,32	-2.883 to 3.523	No	ns	>0.9999
HFCN vs. HFBN	0,09	-3.113 to 3.293	No	ns	>0.9999
HFB vs. HFBN	-0,23	-3.433 to 2.973	No	ns	>0.9999
