# Supplementary material for: Dietary pH Enhancement Improves Metabolic Outcomes in Diet-Induced Obese Male and Female Mice: Effects of Beef vs. Casein Proteins
Source: Nutrients. 2022 Jun 22;14(13):2583. doi: 10.3390/nu14132583 (PMC9268221; doi:10.3390/nu14132583)
Supplement: Supplementary file 1 [file nutrients-14-02583-s001.zip › Supplementary Table S3A_Male GTT stats of each time point.pdf]

Supplementary Table S3A: Male glucose tolerance test results (Time point 0 to 120 minutes). Legends: LFC, low fat casein; LFCN, low fat casein pH enhanced; LFB, low fat beef; LFBN, low fat beef pH enhanced; HFC, high fat casein; HFCN, high fat casein pH enhanced; HFB, high fat beef; HFBN, high fat beef pH enhanced.

Male GTT 0 Minute:

Tukey's multiple comparisons test	Mean Diff.	95.00% CI of diff.	Below threshold?	Summary	Adjusted P Value
LF vs. LFN	-6,2	-47.13 to 34.73	No	ns	0,9997
LF vs. LFB	-32,1	-73.03 to 8.830	No	ns	0,2345
LF vs. LFBN	-20,7	-61.63 to 20.23	No	ns	0,7607
LF vs. HF	-44,7	-85.63 to -3.770	Yes	*	0,0226
LF vs. HFN	-52,7	-93.63 to -11.77	Yes	**	0,0034
LF vs. HFB	-75,83	-117.9 to -33.78	Yes	****	<0.0001
LF vs. HFBN	-51,4	-92.33 to -10.47	Yes	**	0,0047
LFN vs. LFB	-25,9	-66.83 to 15.03	No	ns	0,5048
LFN vs. LFBN	-14,5	-55.43 to 26.43	No	ns	0,9534
LFN vs. HF	-38,5	-79.43 to 2.430	No	ns	0,0802
LFN vs. HFN	-46,5	-87.43 to -5.570	Yes	*	0,0151
LFN vs. HFB	-69,63	-111.7 to -27.58	Yes	****	<0.0001
LFN vs. HFBN	-45,2	-86.13 to -4.270	Yes	*	0,0202
LFB vs. LFBN	11,4	-29.53 to 52.33	No	ns	0,9878
LFB vs. HF	-12,6	-53.53 to 28.33	No	ns	0,9783
LFB vs. HFN	-20,6	-61.53 to 20.33	No	ns	0,7651
LFB vs. HFB	-43,73	-85.78 to -1.682	Yes	*	0,0357
LFB vs. HFBN	-19,3	-60.23 to 21.63	No	ns	0,8191
LFBN vs. HF	-24	-64.93 to 16.93	No	ns	0,6014
LFBN vs. HFN	-32	-72.93 to 8.930	No	ns	0,238
LFBN vs. HFB	-55,13	-97.18 to -13.08	Yes	**	0,0027
LFBN vs. HFBN	-30,7	-71.63 to 10.23	No	ns	0,2859
HF vs. HFN	-8	-48.93 to 32.93	No	ns	0,9986
HF vs. HFB	-31,13	-73.18 to 10.92	No	ns	0,3017
HF vs. HFBN	-6,7	-47.63 to 34.23	No	ns	0,9996
HFN vs. HFB	-23,13	-65.18 to 18.92	No	ns	0,6756
HFN vs. HFBN	1,3	-39.63 to 42.23	No	ns	>0.9999
HFB vs. HFBN	24,43	-17.62 to 66.48	No	ns	0,6125

Male GTT 30 Minutes:

Tukey's multiple comparisons test	Mean Diff.	95.00% CI of diff.	Below threshold?	Summary	Adjusted P Value
LF vs. LFN	-95,7	-224.0 to 32.59	No	ns	0,2919
LF vs. LFB	-78,05	-214.1 to 58.02	No	ns	0,6268
LF vs. LFBN	-45,8	-174.1 to 82.49	No	ns	0,9512
LF vs. HF	-168,1	-296.4 to -39.81	Yes	**	0,0027
LF vs. HFN	-161,8	-290.1 to -33.51	Yes	**	0,0045
LF vs. HFB	-193,5	-325.3 to -61.72	Yes	***	0,0005
LF vs. HFBN	-180,1	-308.4 to -51.81	Yes	**	0,001
LFN vs. LFB	17,65	-118.4 to 153.7	No	ns	>0.9999
LFN vs. LFBN	49,9	-78.39 to 178.2	No	ns	0,9245
LFN vs. HF	-72,4	-200.7 to 55.89	No	ns	0,6458
LFN vs. HFN	-66,1	-194.4 to 62.19	No	ns	0,7424
LFN vs. HFB	-97,82	-229.6 to 33.98	No	ns	0,2981
LFN vs. HFBN	-84,4	-212.7 to 43.89	No	ns	0,4529
LFB vs. LFBN	32,25	-103.8 to 168.3	No	ns	0,9954
LFB vs. HF	-90,05	-226.1 to 46.02	No	ns	0,4451
LFB vs. HFN	-83,75	-219.8 to 52.32	No	ns	0,5397
LFB vs. HFB	-115,5	-254.9 to 23.92	No	ns	0,1776
LFB vs. HFBN	-102,1	-238.1 to 34.02	No	ns	0,2855
LFBN vs. HF	-122,3	-250.6 to 5.988	No	ns	0,0726
LFBN vs. HFN	-116	-244.3 to 12.29	No	ns	0,1052
LFBN vs. HFB	-147,7	-279.5 to -15.92	Yes	*	0,0175
LFBN vs. HFBN	-134,3	-262.6 to -6.012	Yes	*	0,0337
HF vs. HFN	6,3	-122.0 to 134.6	No	ns	>0.9999
HF vs. HFB	-25,42	-157.2 to 106.4	No	ns	0,9987
HF vs. HFBN	-12	-140.3 to 116.3	No	ns	>0.9999
HFN vs. HFB	-31,72	-163.5 to 100.1	No	ns	0,9949
HFN vs. HFBN	-18,3	-146.6 to 110.0	No	ns	0,9998
HFB vs. HFBN	13,42	-118.4 to 145.2	No	ns	>0.9999

Male GTT 60 Minutes:

Tukey's multiple comparisons test	Mean Diff.	95.00% CI of diff.	Below threshold?	Summary	Adjusted P Value
LF vs. LFN	-32,5	-135.4 to 70.35	No	ns	0,9749
LF vs. LFB	-57,8	-160.7 to 45.05	No	ns	0,6517
LF vs. LFBN	0,3	-102.6 to 103.2	No	ns	>0.9999
LF vs. HF	-147,7	-250.6 to -44.85	Yes	***	0,0007
LF vs. HFN	-120,5	-223.4 to -17.65	Yes	*	0,0108
LF vs. HFB	-171,7	-277.4 to -66.06	Yes	****	<0.0001
LF vs. HFBN	-115	-217.9 to -12.15	Yes	*	0,0179
LFN vs. LFB	-25,3	-128.2 to 77.55	No	ns	0,9942
LFN vs. LFBN	32,8	-70.05 to 135.7	No	ns	0,9736
LFN vs. HF	-115,2	-218.1 to -12.35	Yes	*	0,0175
LFN vs. HFN	-88	-190.9 to 14.85	No	ns	0,1485
LFN vs. HFB	-139,2	-244.9 to -33.56	Yes	**	0,0025
LFN vs. HFBN	-82,5	-185.4 to 20.35	No	ns	0,2103
LFB vs. LFBN	58,1	-44.75 to 161.0	No	ns	0,6457
LFB vs. HF	-89,9	-192.8 to 12.95	No	ns	0,1307
LFB vs. HFN	-62,7	-165.6 to 40.15	No	ns	0,5529
LFB vs. HFB	-113,9	-219.6 to -8.262	Yes	*	0,0256
LFB vs. HFBN	-57,2	-160.1 to 45.65	No	ns	0,6635
LFBN vs. HF	-148	-250.9 to -45.15	Yes	***	0,0007
LFBN vs. HFN	-120,8	-223.7 to -17.95	Yes	*	0,0105
LFBN vs. HFB	-172	-277.7 to -66.36	Yes	****	<0.0001
LFBN vs. HFBN	-115,3	-218.2 to -12.45	Yes	*	0,0174
HF vs. HFN	27,2	-75.65 to 130.1	No	ns	0,991
HF vs. HFB	-24,03	-129.7 to 81.64	No	ns	0,9964
HF vs. HFBN	32,7	-70.15 to 135.6	No	ns	0,974
HFN vs. HFB	-51,23	-156.9 to 54.44	No	ns	0,7973
HFN vs. HFBN	5,5	-97.35 to 108.4	No	ns	>0.9999
HFB vs. HFBN	56,73	-48.94 to 162.4	No	ns	0,7019

Male GTT 90 Minutes:

Tukey's multiple comparisons test	Mean Diff.	95.00% CI of diff.	Below threshold?	Summary	Adjusted P Value
LF vs. LFN	-2,4	-90.06 to 85.26	No	ns	>0.9999
LF vs. LFB	-85,7	-173.4 to 1.955	No	ns	0,0599
LF vs. LFBN	-39,9	-127.6 to 47.76	No	ns	0,8439
LF vs. HF	-199,2	-286.9 to -111.5	Yes	****	<0.0001
LF vs. HFN	-114,5	-202.2 to -26.84	Yes	**	0,0028
LF vs. HFB	-180,8	-273.7 to -87.78	Yes	****	<0.0001
LF vs. HFBN	-127,2	-214.9 to -39.54	Yes	***	0,0006
LFN vs. LFB	-83,3	-171.0 to 4.355	No	ns	0,0744
LFN vs. LFBN	-37,5	-125.2 to 50.16	No	ns	0,8815
LFN vs. HF	-196,8	-284.5 to -109.1	Yes	****	<0.0001
LFN vs. HFN	-112,1	-199.8 to -24.44	Yes	**	0,0037
LFN vs. HFB	-178,4	-271.3 to -85.38	Yes	****	<0.0001
LFN vs. HFBN	-124,8	-212.5 to -37.14	Yes	***	0,0008
LFB vs. LFBN	45,8	-41.86 to 133.5	No	ns	0,7292
LFB vs. HF	-113,5	-201.2 to -25.84	Yes	**	0,0032
LFB vs. HFN	-28,8	-116.5 to 58.86	No	ns	0,9688
LFB vs. HFB	-95,05	-188.0 to -2.077	Yes	*	0,0415
LFB vs. HFBN	-41,5	-129.2 to 46.16	No	ns	0,8157
LFBN vs. HF	-159,3	-247.0 to -71.64	Yes	****	<0.0001
LFBN vs. HFN	-74,6	-162.3 to 13.06	No	ns	0,153
LFBN vs. HFB	-140,9	-233.8 to -47.88	Yes	***	0,0003
LFBN vs. HFBN	-87,3	-175.0 to 0.3554	No	ns	0,0517
HF vs. HFN	84,7	-2.955 to 172.4	No	ns	0,0656
HF vs. HFB	18,45	-74.52 to 111.4	No	ns	0,9985
HF vs. HFBN	72	-15.66 to 159.7	No	ns	0,186
HFN vs. HFB	-66,25	-159.2 to 26.72	No	ns	0,3492
HFN vs. HFBN	-12,7	-100.4 to 74.96	No	ns	0,9998
HFB vs. HFBN	53,55	-39.42 to 146.5	No	ns	0,6224

Male GTT 120 Minutes:

Tukey's multiple comparisons test	Mean Diff.	95.00% CI of diff.	Below threshold?	Summary	Adjusted P Value
LF vs. LFN	14,5	-54.40 to 83.40	No	ns	0,9978
LF vs. LFB	-53,8	-122.7 to 15.10	No	ns	0,2392
LF vs. LFBN	-24,7	-93.60 to 44.20	No	ns	0,9503
LF vs. HF	-187	-255.9 to -118.1	Yes	****	<0.0001
LF vs. HFN	-81,4	-150.3 to -12.50	Yes	**	0,0099
LF vs. HFB	-120,2	-193.3 to -47.09	Yes	****	<0.0001
LF vs. HFBN	-101,7	-170.6 to -32.80	Yes	***	0,0004
LFN vs. LFB	-68,3	-137.2 to 0.6046	No	ns	0,0537
LFN vs. LFBN	-39,2	-108.1 to 29.70	No	ns	0,637
LFN vs. HF	-201,5	-270.4 to -132.6	Yes	****	<0.0001
LFN vs. HFN	-95,9	-164.8 to -27.00	Yes	**	0,0011
LFN vs. HFB	-134,7	-207.8 to -61.59	Yes	****	<0.0001
LFN vs. HFBN	-116,2	-185.1 to -47.30	Yes	****	<0.0001
LFB vs. LFBN	29,1	-39.80 to 98.00	No	ns	0,8884
LFB vs. HF	-133,2	-202.1 to -64.30	Yes	****	<0.0001
LFB vs. HFN	-27,6	-96.50 to 41.30	No	ns	0,9131
LFB vs. HFB	-66,38	-139.5 to 6.709	No	ns	0,1022
LFB vs. HFBN	-47,9	-116.8 to 21.00	No	ns	0,381
LFBN vs. HF	-162,3	-231.2 to -93.40	Yes	****	<0.0001
LFBN vs. HFN	-56,7	-125.6 to 12.20	No	ns	0,1842
LFBN vs. HFB	-95,48	-168.6 to -22.39	Yes	**	0,0028
LFBN vs. HFBN	-77	-145.9 to -8.095	Yes	*	0,018
HF vs. HFN	105,6	36.70 to 174.5	Yes	***	0,0002
HF vs. HFB	66,83	-6.259 to 139.9	No	ns	0,0977
HF vs. HFBN	85,3	16.40 to 154.2	Yes	**	0,0057
HFN vs. HFB	-38,78	-111.9 to 34.31	No	ns	0,7138
HFN vs. HFBN	-20,3	-89.20 to 48.60	No	ns	0,9831
HFB vs. HFBN	18,48	-54.61 to 91.56	No	ns	0,9931
