# Supplementary material for: Dietary pH Enhancement Improves Metabolic Outcomes in Diet-Induced Obese Male and Female Mice: Effects of Beef vs. Casein Proteins
Source: Nutrients. 2022 Jun 22;14(13):2583. doi: 10.3390/nu14132583 (PMC9268221; doi:10.3390/nu14132583)
Supplement: Supplementary file 1 [file nutrients-14-02583-s001.zip › Supplementary Table S3B_Female GTT stats of each time point.pdf]

Supplementary Table S3B: Female glucose tolerance test results (Time point 0 to 120 minutes).
Legends: LFC, low fat casein; LFCN, low fat casein pH enhanced; LFB, low fat beef; LFBN, low fat beef pH enhanced; HFC, high fat casein; HFCN, high fat casein pH enhanced; HFB, high fat beef; HFBN, high fat beef pH enhanced.

Female GTT 0 Minute:

Tukey's multiple comparisons test	Mean Diff.	95.00% CI of diff.	Below threshold?	Summary	Adjusted P Value
LF vs. LFN	-23,9	-61.07 to 13.27	No	ns	0,4844
LF vs. LFB	-27,1	-64.27 to 10.07	No	ns	0,321
LF vs. LFBN	-21,5	-58.67 to 15.67	No	ns	0,6183
LF vs. HF	-44,6	-81.77 to -7.429	Yes	**	0,0082
LF vs. HFN	-32,2	-69.37 to 4.971	No	ns	0,1381
LF vs. HFB	-37,5	-74.67 to -0.3294	Yes	*	0,0465
LF vs. HFBN	-33,9	-71.07 to 3.271	No	ns	0,0995
LFN vs. LFB	-3,2	-40.37 to 33.97	No	ns	>0.9999
LFN vs. LFBN	2,4	-34.77 to 39.57	No	ns	>0.9999
LFN vs. HF	-20,7	-57.87 to 16.47	No	ns	0,6625
LFN vs. HFN	-8,3	-45.47 to 28.87	No	ns	0,9968
LFN vs. HFB	-13,6	-50.77 to 23.57	No	ns	0,945
LFN vs. HFBN	-10	-47.17 to 27.17	No	ns	0,9901
LFB vs. LFBN	5,6	-31.57 to 42.77	No	ns	0,9998
LFB vs. HF	-17,5	-54.67 to 19.67	No	ns	0,8207
LFB vs. HFN	-5,1	-42.27 to 32.07	No	ns	0,9999
LFB vs. HFB	-10,4	-47.57 to 26.77	No	ns	0,9875
LFB vs. HFBN	-6,8	-43.97 to 30.37	No	ns	0,9991
LFBN vs. HF	-23,1	-60.27 to 14.07	No	ns	0,5287
LFBN vs. HFN	-10,7	-47.87 to 26.47	No	ns	0,9853
LFBN vs. HFB	-16	-53.17 to 21.17	No	ns	0,8787
LFBN vs. HFBN	-12,4	-49.57 to 24.77	No	ns	0,9663
HF vs. HFN	12,4	-24.77 to 49.57	No	ns	0,9663
HF vs. HFB	7,1	-30.07 to 44.27	No	ns	0,9988
HF vs. HFBN	10,7	-26.47 to 47.87	No	ns	0,9853
HFN vs. HFB	-5,3	-42.47 to 31.87	No	ns	0,9998
HFN vs. HFBN	-1,7	-38.87 to 35.47	No	ns	>0.9999
HFB vs. HFBN	3,6	-33.57 to 40.77	No	ns	>0.9999

Female GTT 30 Minutes:

Tukey's multiple comparisons test	Mean Diff.	95.00% CI of diff.	Below threshold?	Summary	Adjusted P Value
LF vs. LFN	-101,2	-213.2 to 10.77	No	ns	0,1054
LF vs. LFB	3,878	-111.2 to 118.9	No	ns	>0.9999
LF vs. LFBN	-3,5	-115.5 to 108.5	No	ns	>0.9999
LF vs. HF	-168,5	-283.5 to -53.42	Yes	***	0,0005
LF vs. HFN	-109,8	-224.8 to 5.249	No	ns	0,072
LF vs. HFB	-231,2	-343.2 to -119.2	Yes	****	<0.0001
LF vs. HFBN	-118,9	-233.9 to -3.862	Yes	*	0,0378
LFN vs. LFB	105,1	-9.960 to 220.1	No	ns	0,0982
LFN vs. LFBN	97,7	-14.27 to 209.7	No	ns	0,1318
LFN vs. HF	-67,26	-182.3 to 47.78	No	ns	0,6035
LFN vs. HFN	-8,589	-123.6 to 106.4	No	ns	>0.9999
LFN vs. HFB	-130	-242.0 to -18.03	Yes	*	0,012
LFN vs. HFBN	-17,7	-132.7 to 97.34	No	ns	0,9997
LFB vs. LFBN	-7,378	-122.4 to 107.7	No	ns	>0.9999
LFB vs. HF	-172,3	-290.4 to -54.31	Yes	***	0,0005
LFB vs. HFN	-113,7	-231.7 to 4.360	No	ns	0,0673
LFB vs. HFB	-235,1	-350.1 to -120.0	Yes	****	<0.0001
LFB vs. HFBN	-122,8	-240.8 to -4.751	Yes	*	0,0357
LFBN vs. HF	-165	-280.0 to -49.92	Yes	***	0,0007
LFBN vs. HFN	-106,3	-221.3 to 8.749	No	ns	0,0908
LFBN vs. HFB	-227,7	-339.7 to -115.7	Yes	****	<0.0001
LFBN vs. HFBN	-115,4	-230.4 to -0.3621	Yes	*	0,0487
HF vs. HFN	58,67	-59.36 to 176.7	No	ns	0,775
HF vs. HFB	-62,74	-177.8 to 52.29	No	ns	0,6836
HF vs. HFBN	49,56	-68.47 to 167.6	No	ns	0,8908
HFN vs. HFB	-121,4	-236.4 to -6.373	Yes	*	0,0313
HFN vs. HFBN	-9,111	-127.1 to 108.9	No	ns	>0.9999
HFB vs. HFBN	112,3	-2.738 to 227.3	No	ns	0,0606

Female GTT 60 Minutes:

Tukey's multiple comparisons test	Mean Diff.	95.00% CI of diff.	Below threshold?	Summary	Adjusted P Value
LF vs. LFN	-1,7	-98.03 to 94.63	No	ns	>0.9999
LF vs. LFB	-17,1	-113.4 to 79.23	No	ns	0,9993
LF vs. LFBN	18,2	-78.13 to 114.5	No	ns	0,9989
LF vs. HF	-86,3	-182.6 to 10.03	No	ns	0,112
LF vs. HFN	-58,2	-154.5 to 38.13	No	ns	0,5648
LF vs. HFB	-135,6	-231.9 to -39.27	Yes	***	0,0009
LF vs. HFBN	-46,5	-142.8 to 49.83	No	ns	0,8013
LFN vs. LFB	-15,4	-111.7 to 80.93	No	ns	0,9996
LFN vs. LFBN	19,9	-76.43 to 116.2	No	ns	0,9981
LFN vs. HF	-84,6	-180.9 to 11.73	No	ns	0,127
LFN vs. HFN	-56,5	-152.8 to 39.83	No	ns	0,6015
LFN vs. HFB	-133,9	-230.2 to -37.57	Yes	**	0,0011
LFN vs. HFBN	-44,8	-141.1 to 51.53	No	ns	0,8297
LFB vs. LFBN	35,3	-61.03 to 131.6	No	ns	0,9446
LFB vs. HF	-69,2	-165.5 to 27.13	No	ns	0,3396
LFB vs. HFN	-41,1	-137.4 to 55.23	No	ns	0,8835
LFB vs. HFB	-118,5	-214.8 to -22.17	Yes	**	0,0061
LFB vs. HFBN	-29,4	-125.7 to 66.93	No	ns	0,9794
LFBN vs. HF	-104,5	-200.8 to -8.169	Yes	*	0,0241
LFBN vs. HFN	-76,4	-172.7 to 19.93	No	ns	0,2225
LFBN vs. HFB	-153,8	-250.1 to -57.47	Yes	***	0,0001
LFBN vs. HFBN	-64,7	-161.0 to 31.63	No	ns	0,4269
HF vs. HFN	28,1	-68.23 to 124.4	No	ns	0,9841
HF vs. HFB	-49,3	-145.6 to 47.03	No	ns	0,7501
HF vs. HFBN	39,8	-56.53 to 136.1	No	ns	0,8997
HFN vs. HFB	-77,4	-173.7 to 18.93	No	ns	0,2087
HFN vs. HFBN	11,7	-84.63 to 108.0	No	ns	>0.9999
HFB vs. HFBN	89,1	-7.231 to 185.4	No	ns	0,0904

Female GTT 90 Minutes:

Tukey's multiple comparisons test	Mean Diff.	95.00% CI of diff.	Below threshold?	Summary	Adjusted P Value
LF vs. LFN	4,6	-41.79 to 50.99	No	ns	>0.9999
LF vs. LFB	-31,6	-77.99 to 14.79	No	ns	0,4084
LF vs. LFBN	-10	-56.39 to 36.39	No	ns	0,9975
LF vs. HF	-57,5	-103.9 to -11.11	Yes	**	0,0055
LF vs. HFN	-27,9	-74.29 to 18.49	No	ns	0,5705
LF vs. HFB	-54,6	-101.0 to -8.209	Yes	*	0,0102
LF vs. HFBN	-29,8	-76.19 to 16.59	No	ns	0,4856
LFN vs. LFB	-36,2	-82.59 to 10.19	No	ns	0,2403
LFN vs. LFBN	-14,6	-60.99 to 31.79	No	ns	0,9755
LFN vs. HF	-62,1	-108.5 to -15.71	Yes	**	0,002
LFN vs. HFN	-32,5	-78.89 to 13.89	No	ns	0,3718
LFN vs. HFB	-59,2	-105.6 to -12.81	Yes	**	0,0038
LFN vs. HFBN	-34,4	-80.79 to 11.99	No	ns	0,3001
LFB vs. LFBN	21,6	-24.79 to 67.99	No	ns	0,8288
LFB vs. HF	-25,9	-72.29 to 20.49	No	ns	0,6596
LFB vs. HFN	3,7	-42.69 to 50.09	No	ns	>0.9999
LFB vs. HFB	-23	-69.39 to 23.39	No	ns	0,7789
LFB vs. HFBN	1,8	-44.59 to 48.19	No	ns	>0.9999
LFBN vs. HF	-47,5	-93.89 to -1.109	Yes	*	0,041
LFBN vs. HFN	-17,9	-64.29 to 28.49	No	ns	0,928
LFBN vs. HFB	-44,6	-90.99 to 1.791	No	ns	0,0682
LFBN vs. HFBN	-19,8	-66.19 to 26.59	No	ns	0,8834
HF vs. HFN	29,6	-16.79 to 75.99	No	ns	0,4945
HF vs. HFB	2,9	-43.49 to 49.29	No	ns	>0.9999
HF vs. HFBN	27,7	-18.69 to 74.09	No	ns	0,5795
HFN vs. HFB	-26,7	-73.09 to 19.69	No	ns	0,6243
HFN vs. HFBN	-1,9	-48.29 to 44.49	No	ns	>0.9999
HFB vs. HFBN	24,8	-21.59 to 71.19	No	ns	0,7068

Female GTT 120 Minutes:

Tukey's multiple comparisons test	Mean Diff.	95.00% CI of diff.	Below threshold?	Summary	Adjusted P Value
LF vs. LFN	-0,7	-34.60 to 33.20	No	ns	>0.9999
LF vs. LFB	-41,5	-75.40 to -7.601	Yes	**	0,0065
LF vs. LFBN	-17,08	-51.91 to 17.75	No	ns	0,7879
LF vs. HF	-43,3	-77.20 to -9.401	Yes	**	0,0038
LF vs. HFN	-32	-65.90 to 1.899	No	ns	0,0781
LF vs. HFB	-45,6	-79.50 to -11.70	Yes	**	0,0019
LF vs. HFBN	-28,2	-62.10 to 5.699	No	ns	0,1739
LFN vs. LFB	-40,8	-74.70 to -6.901	Yes	**	0,0079
LFN vs. LFBN	-16,38	-51.21 to 18.45	No	ns	0,8212
LFN vs. HF	-42,6	-76.50 to -8.701	Yes	**	0,0047
LFN vs. HFN	-31,3	-65.20 to 2.599	No	ns	0,0914
LFN vs. HFB	-44,9	-78.80 to -11.00	Yes	**	0,0023
LFN vs. HFBN	-27,5	-61.40 to 6.399	No	ns	0,1986
LFB vs. LFBN	24,42	-10.41 to 59.25	No	ns	0,3702
LFB vs. HF	-1,8	-35.70 to 32.10	No	ns	>0.9999
LFB vs. HFN	9,5	-24.40 to 43.40	No	ns	0,9874
LFB vs. HFB	-4,1	-38.00 to 29.80	No	ns	>0.9999
LFB vs. HFBN	13,3	-20.60 to 47.20	No	ns	0,9216
LFBN vs. HF	-26,22	-61.05 to 8.606	No	ns	0,2814
LFBN vs. HFN	-14,92	-49.75 to 19.91	No	ns	0,881
LFBN vs. HFB	-28,52	-63.35 to 6.306	No	ns	0,1891
LFBN vs. HFBN	-11,12	-45.95 to 23.71	No	ns	0,9734
HF vs. HFN	11,3	-22.60 to 45.20	No	ns	0,9663
HF vs. HFB	-2,3	-36.20 to 31.60	No	ns	>0.9999
HF vs. HFBN	15,1	-18.80 to 49.00	No	ns	0,8583
HFN vs. HFB	-13,6	-47.50 to 20.30	No	ns	0,9126
HFN vs. HFBN	3,8	-30.10 to 37.70	No	ns	>0.9999
HFB vs. HFBN	17,4	-16.50 to 51.30	No	ns	0,7469
